# Histidine-51 Facilitates the Conformational Change and Deprotonation of Horse Liver Alcohol Dehydrogenase–NAD^+^ Complexes

**DOI:** 10.1021/acs.biochem.6c00532

**Published:** 2026-10-22

**Authors:** Bryce V. Plapp

**Affiliations:** Department of Biochemistry and Molecular Biology, The University of Iowa, Bowen Science Building 51 Newton Road, Iowa City, Iowa 52242-1109, United States

## Abstract

Structural studies suggest that His-51 in wild-type horse liver alcohol dehydrogenase (ADH1E) facilitates proton transfer between the hydroxyl group of a ligand bound to the catalytic zinc and bulk solvent. Previous work showed that the Gln-51 substitution increases the p*K* values for pH dependencies for various enzymatic reactions by about 1.5 units, and stopped-flow studies on the binding of NAD^+^ and pyrazole suggested that the equilibrium position for the global conformational change of the enzyme–NAD^+^ complex became disfavored by 1000-fold. That conclusion needed confirmation. Here, X–ray crystallography of six ternary complexes of H51Q ADH with NAD^+^ and pyrazole, caprate, 2,2,2-trifluoroethanol, or 2,3,4,5,6-pentafluorobenzyl alcohol, and NADH and water or *N*-cyclohexylformamide shows that all complexes have the same closed conformation as wild-type ADH. Gln-51 fits in the region occupied by His-51 and does not affect the positions of bound coenzymes or inhibitors that coordinate to the catalytic zinc. Stopped-flow studies show that the rate-limiting formation of ternary complexes with H51Q ADH and NAD^+^ depends upon deprotonation of the ligands that bind to the catalytic zinc (pyrazole or 2,2,2-trifluoroethanol). Furthermore, the binding of NAD^+^ and trifluoroethanol monitored by fluorescence and proton release show that the H51Q substitution disfavors the conformational change by about 3-fold as compared to wild-type ADH. The p*K* values of the enzyme–NAD^+^ complexes with water and other zinc ligands increase because His-51 no longer facilitates the deprotonation. The roles of the zinc-water and His-51 in the mechanism are exposed.

## Graphical abstract

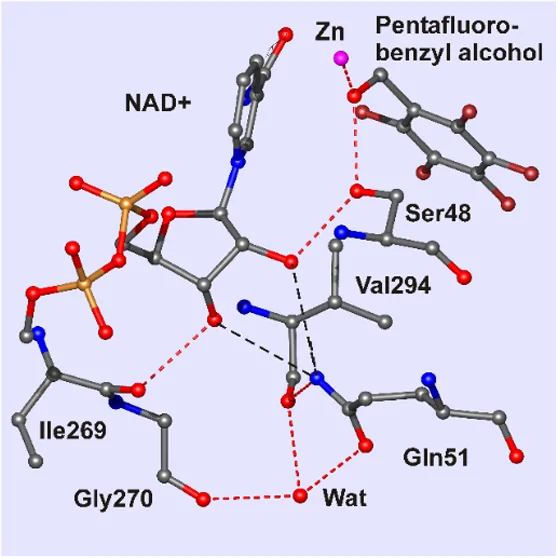

## Introduction

Horse liver alcohol dehydrogenase (ADH1E, EC 0.1.1.1.1) catalyzes proton and hydride transfers in the reversible oxidation of alcohols. ADH has a predominantly ordered mechanism that has a kinetically and structurally characterized conformational change when NAD^+^ binds (Scheme 1).^1–9^ The enzyme–NADH complex also has a closed conformation, as determined by X-ray crystallography, but the isomerization is very fast and rate constants for the two-step reaction are not well determined.^2,3,7,10,11^ The conformational change when NAD^+^ or NADH bind to the apoenzyme results in quenching of protein fluorescence, which is attributed to energy transfer with Trp-314.^4,5,12–21^ The isomerization (step 2) in the mechanism is pH dependent, and the contributions of His-51 and other ionizable groups in the active site should be evaluated.

**Scheme 1:**
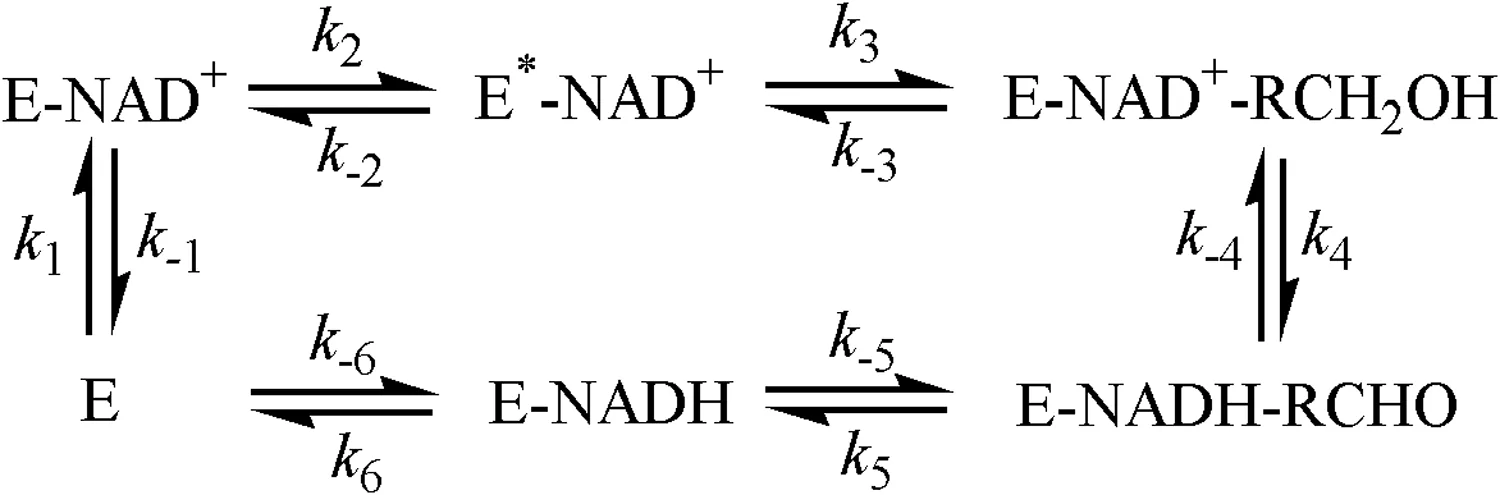
Kinetic Mechanism with Isomerization of the Enzyme–NAD^+^ Complex

Equilibrium binding of NAD^+^ is linked to a group on the apoenzyme with a p*K* of ∼9.6, which is shifted to a p*K* of ∼7.5 in the enzyme–NAD^+^ complex and to a p*K* of ∼5 in ternary complexes with pyrazole or 2,2,2-trifluoroethanol.^11,14,22–25^ NAD^+^ binds most tightly at high pH.^22,25^ The bimolecular rate constant for binding of NAD^+^ decreases as the pH increases with a p*K* of ∼9.2, and the enzyme–NAD^+^ complex dissociates most rapidly at low pH below a p*K* of ∼7.6.^6,11^ The p*K* of 9.2–9.6 is generally attributed to the ionization of the catalytic zinc–water that is shifted to ∼7.5 in the enzyme–NAD^+^ complex because the positively charged nicotinamide ring is close to the zinc–hydroxide.^23,26–30^ Another study showed that the bimolecular rate constant for binding of NAD^+^ has a bell-shaped pH dependence with p*K* values of 6.9 and 9.0 (most rapid binding at pH 8) that were assigned to the zinc-water and Lys-228 from studies of reactions in the presence of imidazole, which replaces the water, or when Lys-228 is chemically modified.^6,31^ However, the chemical interventions seem to hinder the conformational change when coenzymes bind.^31–33^

Transient kinetic studies on the binding of NAD^+^ to wild-type ADH, using absorbance, fluorescence, and proton release signals, showed that the initial ADH–NAD^+^ complex isomerizes and deprotonates with a p*K* of 7.3 before pyrazole, 2,2,2-trifluoroethanol or caprate bind to form ternary complexes.^8^ The p*K* might be assigned to the zinc-water or His-51, which are linked by the proton relay system through a hydrogen-bonded system that includes Ser-48 and a water in the apoenzyme or Ser-48 and the 2′-hydroxyl group of the nicotinamide ribose in complexes with NAD^+^ or NADH (Scheme 2).^8,11,30,34^ Inactivation of the apoenzyme by diethyl pyrocarbonate, which selectively reacts with histidine residues, is most rapid at pH above a p*K* of 9.6, perhaps because His-51 is more reactive when the zinc-water is ionized.^35^ The substitution of His-51 with glutamine (H51Q) shifts the pH dependencies upward for many steps in the mechanism.^36^ It also appeared that the rate of binding of NAD^+^ in the presence of pyrazole decreases by 10-fold at pH 8, that the pH dependence shifts, and that the equilibrium position for the isomerization (“conformational change”) shifts from 10-fold in favor of the closed form to 100-fold in favor of the open conformation at pH 8.^31^ This suggestion needed further examination with experimental signals for the conformational change and proton release with varied ligands that bind to the enzyme–NAD^+^ complex.

**Scheme 2:**
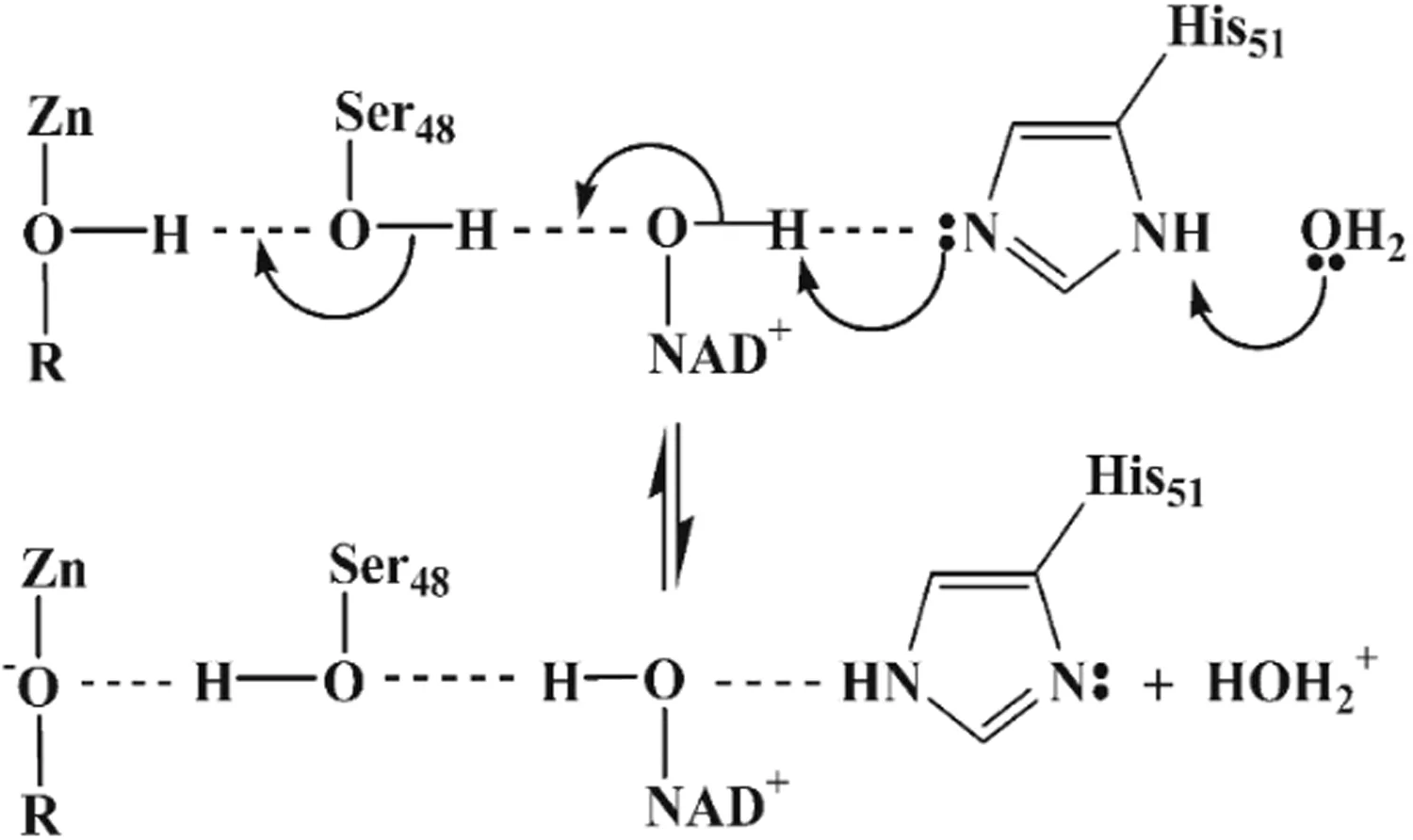
Proton Relay System in Horse Liver ADH with Zinc-Water or Alcohol

Here, the formation of ternary complexes of H51Q ADH with NAD^+^ was studied with stopped-flow kinetics with four different ligands that bind to the catalytic zinc. Proton release and formation of ternary complexes with pyrazole and trifluoroethanol are coincident and rate-limiting. Because the kinetic constants differ for binding of pyrazole and trifluoroethanol or ethanol oxidation, the H51Q substitution affects the formation of the ternary complexes rather than the isomerization where the rate constants should be the same with different ligands. Nevertheless, the conformational change is also affected. The binding of NAD^+^ and caprate to wild-type ADH shows a fast transient proton uptake that is coupled to the conformational change of the enzyme–NAD^+^ complex and a slower release of a proton during the binding of caprate, with no net change in pH. That biphasic transient is greatly diminished with H51Q ADH, indicating altered rate constants or an increased p*K* value of the enzyme–NAD^+^ complex. The binding of NAD^+^ and trifluoroethanol monitored by fluorescence suggests that the H51Q substitution decreases the conformational equilibrium (Scheme 1, *k*_2_/*k*_–2_) by about 3-fold. The previous study, where the complex with NAD^+^ was trapped with pyrazole, suggested a 1000-fold reversal in *k*_2_/*k*_–2_, but that reaction is now shown to be limited by deprotonation of the ternary complex.^31^ Binding of NAD^+^ to H51Q ADH is almost pH independent, and the macroscopic p*K* value for the enzyme–NAD^+^–zinc–water complex is increased by about 1.5 unit. The p*K* values for other ternary complexes also increase, because of the loss of base catalysis by His-51. X-ray crystallography of six different ternary complexes of H51Q ADH shows how the H51Q substitution affects the proton relay system and identifies alternative positions for ligands and amino acid residues that are relevant for understanding the dynamics of catalysis. This study presents a unified explanation of the effects of substituting His-51 on the enzyme reactions and illustrates the value of kinetic simulations of transient data that provide estimates of rate constants for different mechanisms.

## Materials and Methods

### Materials

Li-NAD^+^ (grade I) and Na_2_NADH were obtained from Roche Molecular Biochemicals. Pyrazole, 2,2,2-trifluoroethanol, 2,3,4,5,6-pentafluorobenzyl alcohol, and *N*-cyclohexylformamide were purchased from Aldrich. Sodium caprate was from Fluka Chemika. Phenol red, thymol blue and thymolphthalein sodium salts (ACS reagent grade) were purchased from ICN Biomedicals Inc., and chlorophenol red was from Nutritional Biochemicals Corporation.

### Protein Preparation

The plasmid for expression of the H51Q ADH in *Escherichia coli* XL1–Blue was prepared by mutagenesis of the cDNA for the wild-type horse liver ADH1E (GenBank M64864, Uniprot P00327).^31,36,37^ The enzyme was purified by the published procedure except that 10 mM Tris–HCl, 0.25 mM EDTA, pH 7.9 (4 °C) buffer was used for developing the DEAE–Sepharose column.^37^ The protein was homogeneous on gel electrophoresis and free of contaminating nucleotides,^38^ with an *A*_280_/*A*_260_ ratio of 1.45, and *A*_280_/cm assumed to be 0.455/(mg/mL) as for wild-type ADH. The concentration (N, normality, needed for simulations) of enzyme active sites was determined by titration with NAD^+^ in the presence of pyrazole, and typically 83% of the subunits could bind NAD^+^.^39^

### Transient Kinetics

Transient kinetics were studied with the BioLogic SFM 3 stopped-flow spectrophotometer (dead time 2.4 ms) using varied concentrations of ligands. The binding of NAD^+^ to H51Q ADH was studied in the presence of trapping agents, pyrazole, 2,2,2-trifluoroethanol or caprate in a weakly buffered solution (33 mM Na_2_SO_4_, 0.25 mM EDTA, under N_2_ atmosphere, 25 °C) as described previously.^8^

The reaction of NAD^+^ with pyrazole in the ternary complex is monitored by absorbance at 293 nm.^39,40^ Absorbance at 280 nm reports on the binding of the adenine ring of the NAD^+^, which is enhanced upon binding of caprate.^40,41^ The quenching of protein fluorescence (Fl, typically excitation 290–295 nm, emission 320–340 nm) is associated with the buried Trp-314 and the conformational change that closes up the active site.^5,8,13,16,18,19,21,42,43^ Proton release was monitored with pH indicators at wavelengths in the visible region: chlorophenol red (pH 6.5, 575 nm), phenol red (pH 6.9–8.7, 559 nm), thymol blue (pH 8.6–9.4, 596 nm), thymolphthalein (pH 9.8, 595 nm). The number of protons released per active site of the H51Q enzyme ([H^+^]/[E]) was determined from the amplitudes of transient stopped-flow data as described previously, where the solutions of the enzyme and the indicator were titrated with standardized HCl or NaOH to obtain the practical extinction coefficient and by potentiometric titration with no indicator.^8^

Some reactions were also studied in buffers with 33 mM Na_2_SO_4_, 0.25 mM EDTA, and sodium salts of 10 mM 3-(*N*-morpholino)­propanesulfonic acid (MOPS, pH 6.7, 7.2), *N*-tris­(hydroxymethyl)­methyl-3-aminopropanesulfonic acid (TAPS, pH 8.0, 8.6) or 3-([1,1-dimethyl-2-hydroxyethyl]­amino)-2-hydroxypropanesulfonic acid (AMPSO, pH 8.2, 9.0–9.7).

### Macroscopic Data Analysis

Stopped–flow progress curves were fitted with a function for a single exponential followed by a linear term using the BioKine software for the BioLogic instrument. The apparent kinetic constants *k*_app_ and *k*_app_/*K*_S_ were determined from the fit of the observed rate constants at varied concentrations of NAD^+^ with the Michaelis–Menten equation, *k*_obs_ = *k*_app_[S]/(*K*_S_ + [S]), using the HYPER program.^44^ The observed rate constants for the reaction of the enzyme with varied concentrations of both NAD^+^ and inhibitor (I) were fitted with the equation for a Sequential Bi mechanism, *k*_obs_ = *k*_lim_[NAD^+^]­[I]/(*K*_ia_ *K*_I_ + *K*_NAD_[I] + *K*_I_[NAD^+^] + [NAD^+^]­[I]), using the SEQUEN program or for a Ping Pong mechanism, *k*_obs_ = *k*_lim_[NAD^+^]­[I]/(*K*_NAD_[I] + *K*_I_[NAD^+^] + [NAD^+^]­[I]), with the PINGPONG program.^44^ The nonlinear least-squares analysis program NONLIN was used to fit pH dependencies of the kinetic constants and proton stoichiometry data.^45^

### Simulations of the Transient Kinetic Data

Computer programs that use numerical integration of the differential equations describing the various mechanisms were used to simulate and fit progress curves and estimate microscopic rate constants.^46–50^ For wild-type ADH, global fitting of transient data for binding of NAD^+^ and pyrazole, trifluoroethanol, or caprate with the Kinsim/Fitsim and Dynafit programs provided good estimates of the rate constants for a mechanism involving deprotonation and isomerization of the enzyme–NAD^+^ complex.^8,47,51^ The program KinTek Explorer fit the data well as shown in the “Alcohol Dehydrogenase” example.^48^ Complementary studies provided estimates of rate constants for some steps in the mechanism.^6,7,31,36^ Critical factors in simulations are enzyme concentration, signals with different ligands, output factors for amplitudes, zero time points corrected for the dead-time of the instrument, and baselines. Information about the concentrations of intermediate enzyme forms is required to determine the rate constants, because even the rate of formation of product in the simplest two step mechanism can be described by either fast and slow or slow and fast steps.^52^

The same overall procedures were used for H51Q ADH. Several rounds of simulations led to convergence with small standard errors, no estimates hitting initial limits, good fits (visual and *R* ^2^ > 0.999), and support for the simplest relevant mechanisms. More complex mechanisms cannot be excluded. Estimates of rate constants for some binding steps are highly correlated, but the ratios provided reasonable equilibrium dissociation constants. The kfsim40.exe programs for Kinsim/Fitsim are available from http://www.biochem.wustl.edu/cflab/message.html or the author’s entry in the Departmental Web site: http://bmb.medicine.uiowa.edu These DOS programs run under Windows operating systems with the DOSBox application.

### X-ray Crystallography

Six different complexes of the H51Q enzyme with added ligands were crystallized by dialysis of 10 mg/mL protein in 50 mM ammonium *N*-[tris­(hydroxymethyl)­methyl]-2-aminoethanesulfonate buffer, 0.25 mM EDTA, pH 7.0 at 4 °C (pH 6.7 at 25 °C) by slow addition of 2-methyl-2,4-pentanediol. The crystals were flash vitrified in liquid nitrogen, and X-ray data were collected at 100 K. An R-Axis IV^+2^ with a rotating anode generator provided data to about 1.45 Å resolution for five complexes. High resolution data (1.2 Å) for one complex were collected at IMCA-CAT, APS ID17. The coordinates for the wild-type ADH–NAD^+^–4-iodopyrazole complex (1N92.pdb) or of the V292T ADH–NAD^+^–pyrazole complex (1N8K.pdb) were used for molecular replacement, and the structures were refined by cycles of restrained refinement with REFMAC 5.8.0267.^53^ The REFMAC dictionary for NAD^+^ (NAJ) was modified to use relaxed restraints for the nicotinamide ring so that planarity or puckering for NAD^+^ or with NADH and bond distances could be fitted, such as for the complexes with NAD and pyrazole or with NADH and *N*-cyclohexylformamide or water.^3,54,55^ The final refinements with NADH used a dictionary (NAI) with relaxed restraints on the nicotinamide ring.^3,56^ Structural models were produced with the program O^57^ and analyzed with the MolProbity and Parvati servers.^58,59^ See more details about the crystallography in Supporting Information and Table 1.

**Table 1:** X-ray Data and Refinement Statistics for H51Q ADHs Complexed with NAD­(H) and Substrate Analogues^a^

ligands	PFB, NAD^+^	TFE, NAD(H)	Pyrazole–NAD	Caprate, NAD^+^	*N*-Cyclohex-ylformamide, NADH	Water, NADH
PDB entry	8G4V	8G2L	8G2X	8G2S	8G39	8G41
cell dimensions (Å)	44.2, 51.6, 92.8	44.0, 51.1, 92.6	44.1, 50.8, 92.4	44.2, 51.2, 92.5	44.1,50.9, 92.7	44.1, 51.3, 92.8
cell angles (deg)	92.0, 102.9, 110.0	92.1, 103.0, 109.4	92.1, 103.0, 109.7	92.0, 102.9, 109.9	92.1, 103.0, 109.7	91.9, 103.1, 109.9
mosaicity	1.11	1.46	0.68	0.91	0.97	0.67
resolution range (Å), (shell)	19.96–1.20 (1.24)	19.87–1.42 (1.47)	19.99–1.47 (1.52)	19.98–1.45 (1.50)	19.91–1.42 (1.47)	19.90–1.50 (1.55)
no. of reflections (total, unique)	1298467, 207414	745662, 118680	218163, 110530	433911, 112223	286519, 119503	414050, 105749
redundancy	6.13 (3.50)	6.16 (4.78)	1.95 (1.92)	3.79 (3.55)	2.35 (2.35)	3.84 (3.75)
completeness (%) (outer shell)	90.0 (68.4)	87.2 (51.3)	90.1 (82.9)	87.4 (47.6)	87.8 (66.0)	91.3 (84.3)
*R*_pim_ (%) (outer shell)^b^	2.6 (11.5)	2.5 (19.2)	3.0 (25.9)	1.7 (12.8)	2.4 (21.3)	1.8 (10.2)
average ⟨I⟩/σ⟨I⟩ (outer shell)	13.3 (4.2)	17.2 (2.8)	14.3 (2.5)	23.8 (4.3)	16.0 (2.8)	25.0 (5.7)
*R*_value_, *R*_free_, test (%, number)^c^	13.5, 16.1 1.0 (2091)	18.6, 21.1, 2.0 (2406)	17.3, 19.1, 1.5 (1659)	15.7, 18.9, 2.0 (2272)	14.6. 19.1, 2.0 (2403)	15.5, 17.7. 2.0 (2114)
RMSD for bond distances^d^ (Å)	0.020	0.014	0.013	0.013	0.017	0.014
RMSD for bond angles^d^ (deg)	2.26	1.95	1.88	1.99	2.08	1.94
estimated errors in coordinates (Å)	0.023	0.053	0.047	0.040	0.046	0.042
mean *B*-factor (Å^2^) (Wilson, REFMAC)	11.4, 17.4	19.4, 21.4	11.5, 19.0	12.3, 14.6	15.2, 21.3	11.9, 15.0
non (H atoms, total)^e^	6688	6601	6565	6737	6520	6598
protein (*B*-factors)	5871 (17.7)	5780 (22.5)	5737 (19.4)	5808 (16.3)	5745 (21.8)	5798 (16.0)
4 Zn, 2 NAD^+^, 2 ligands, 2–4 MPD	126 (16.5)	140 (22.3)	126 (19.1)	164 (17.3)	126 (20.6)	124 (16.1)
waters	691 (28.8)	681 (32.4)	782 (29.9)	765 (27.6)	649 (32.4)	676 (25.4)
Ramachandran, % favored (outliers)	97.18 (0)	97.3 (2)	97.18 (1)	97.6 (1)	97.13 (0)	97.45 (1)
MolProbity: clash, score, rank	1.39, 98th; 0.99, 99th	1.49, 99th; 1.03, 99th	1.43. 99th; 1.01, 99th	1.56, 99th; 1.00, 99th	1.76, 99th; 1.04. 99th	0.83, 99th, 0.86, 100th
Matthews coeff., fraction solvent	2.28, 0.46	2.26, 0.45	2.24, 0.45	2.26, 0.46	2.25, 0.45	2.26, 0.47

a The space groups are *P*_1_, with one homodimeric molecule with 748 amino acid residues in the asymmetric unit.

b *R*_pim_, redundancy independent merging, = *R*_merge_/(*n* – 1)^1/2^, where *n* is data redundancy.

c *R*_value_ = (Σ|*F*_0_ *– kF*_c_|)/Σ|*F*_0_|, where *k* is a scale factor. *R*_free_ was calculated with the indicated percentage of reflections not used in the refinement.^60^

d Deviations from ideal geometry.

e The data in the following three lines were calculated with the PARVATI server.^59^

## Results

### Kinetics of Proton Release and Binding of NAD^+^ and Pyrazole

Stopped-flow kinetics were used to study the binding of NAD^+^ to H51Q ADH by trapping the enzyme-NAD^+^ complex with pyrazole, which displaces a water bound to the catalytic zinc, deprotonates, and forms a partial covalent bond between N2 of pyrazole and C4N of the nicotinamide ring.^39,61,62^ The adduct has a broad absorbance maximum at 284–293 nm and a difference extinction coefficient per active site normality of 9200 M^–1^cm^–1^ at 293 nm, which is similar to the value for wild-type enzyme.^8,39^ Pyrazole is an uncompetitive inhibitor against varied concentrations of NAD^+^ and competitive against ethanol, indicating that it binds preferentially to the enzyme-NAD^+^ complex.^63^ Proton release was monitored in parallel reactions by the decrease in absorbance of a pH indicator.

Transient data for each concentration of NAD^+^ fit a single exponential process, and the observed rate constants for both proton release and formation of the enzyme–NAD^+^–pyrazole complex (Figure 1) show saturation kinetics as the concentration of NAD^+^ is increased. The data fit hyperbolic kinetics, and the kinetic constants for binding and proton release are essentially the same with the same concentrations of ligands. The amplitudes of the reaction with pyrazole (*A*_293_) show that the binding eventually goes to completion, and the amplitudes of proton release (*A*_559_) provide the number of protons released per active site.

**Figure 1:**
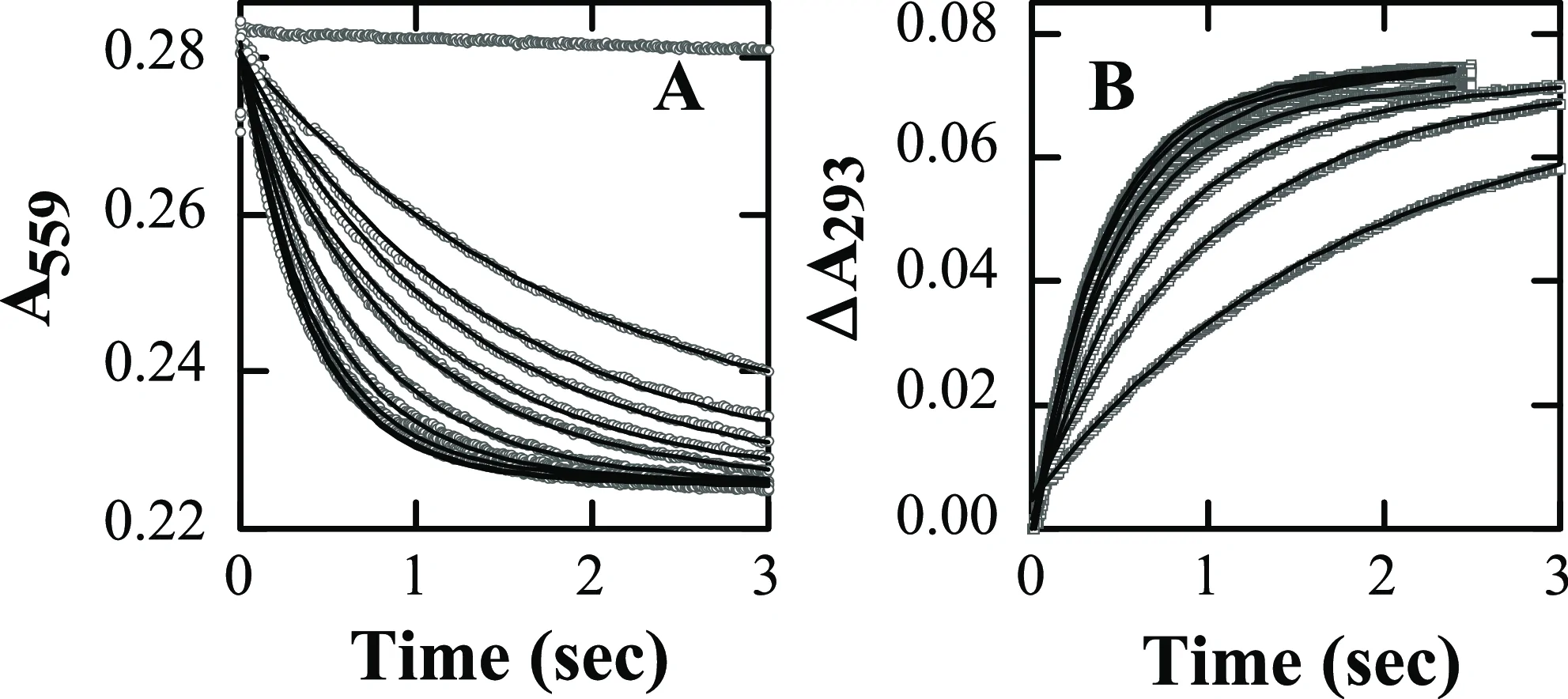
Transient kinetics for proton release and formation of the H51Q ADH–NAD^+^–pyrazole complex. H51Q ADH (10.9 μN) was allowed to react with varied concentrations of NAD^+^ in the presence of and pyrazole (10 mM). (A) Proton release was monitored by a decrease in absorbance of 10 μM phenol red (*A*_559_) with 0, 0.05, 0.07, 0.10, 0.12, 0.15, 0.26, 0.52, 0.78, 0.91, and 1.16 mM NAD^+^ (traces from top to bottom). (B) Formation of the enzyme–NAD^+^–pyrazole (Δ*A*_293_) with 0.07, 0.12, 0.22, 0.52, 0.78, 0.91, and 1.16 mM NAD^+^ (traces from bottom to top). The reactions were in 33 mM Na_2_SO_4_, 0.25 mM EDTA at pH 7.9 and 25 °C under N_2_ atmosphere. Lines are fits for a single exponential reaction and overlap the data points.

Similar results were obtained with varied pH. A fixed concentration of 10 mM pyrazole was used in order to be able to compare to the data for wild-type ADH,^6,8^ and therefore the kinetic constants are labeled as “apparent” (*app*) in Supporting Information Table S1. The kinetic constants (*k*_app_ and *k*_app_/*K*_NAD_) for proton release and formation of the ternary complex with H51Q ADH are strongly dependent on pH in the range from 6.5 to 9.8 with faster rate constants as pH increases above p*K* values of 9–10 (Figure 2A, Supporting Information Table S2). The congruence of the pH profiles indicates that proton release is kinetically coupled to the rate-limiting step in the formation of the enzyme–NAD^+^–pyrazole complex, as shown previously for reaction of wild-type ADH (Figure 2B). However, the pH dependencies for the reactions with wild-type or H51Q ADHs clearly differ in magnitude and direction. The H51Q substitution significantly decreases proton release and the rate-limiting steps for the formation of the enzyme–NAD^+^–pyrazole complex at lower pH values. The present results confirm the pH dependence for binding of NAD^+^ and pyrazole that was found for H51Q and H51Q/K228R ADHs in phosphate/pyrophosphate buffers, and now proton release is identified as a rate-limiting step.^31^ The H51Q substitution has a large effect on the formation of the ternary complex, but the mechanism needs to be explained.

**Figure 2:**
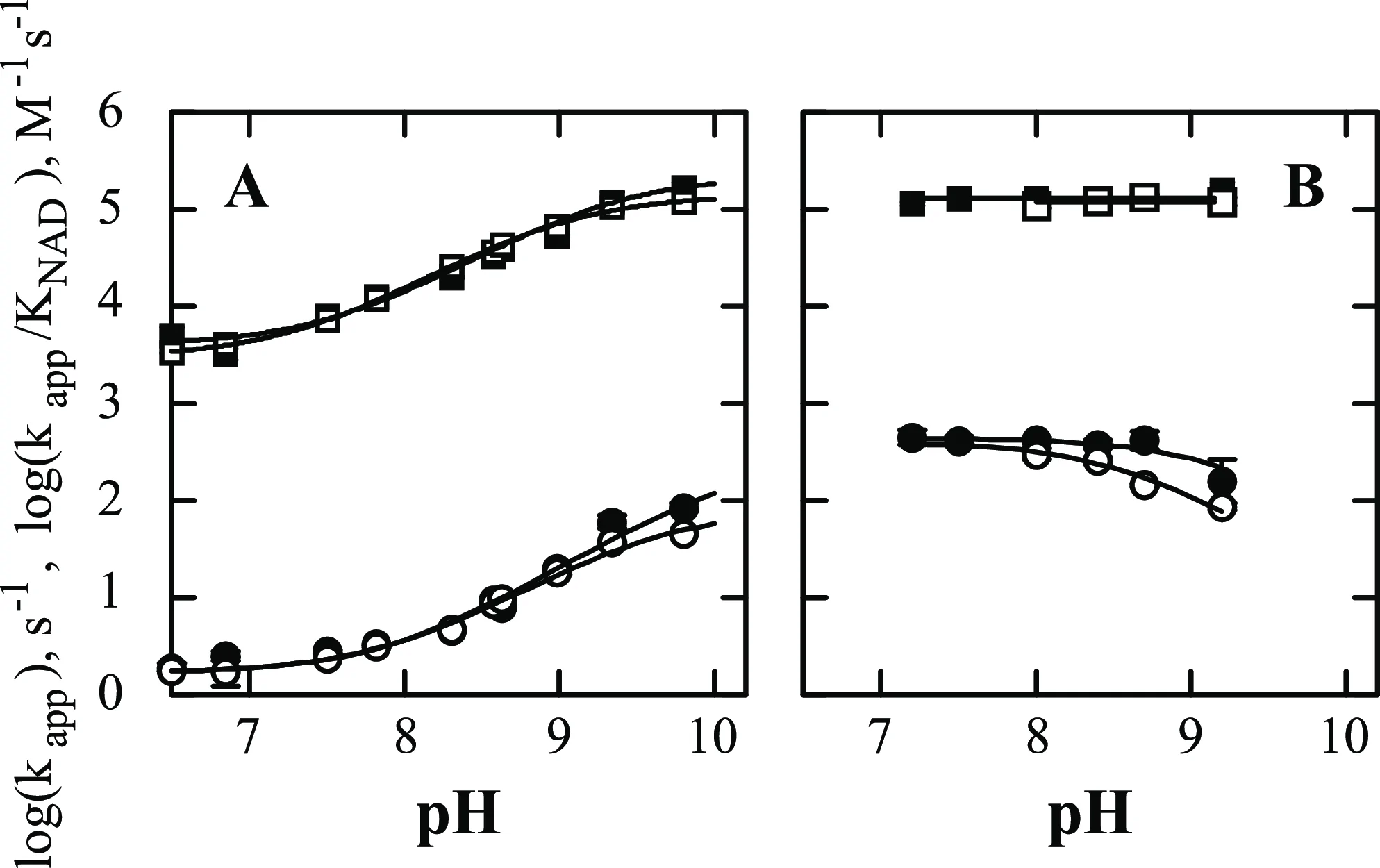
pH dependence of the kinetic constants for transient proton release and for formation of the H51Q ADH–NAD^+^–pyrazole complex. The apparent kinetic constants for proton release (*k*_app_, ● *k*_app_/*K*_NAD_, ■) and for formation of the enzyme–NAD^+^–pyrazole complex (*k*_app_, ○ *k*_app_/*K*_NAD_, □) in reactions of (A) the H51Q and (B) the wild-type enzymes with NAD^+^ and 10 mM pyrazole. The data for H51Q ADH are given in Supporting Information Table S1. The p*K* values for the pH dependencies for H51Q ADH are given in Supporting Information Table S2. Data for the wild-type ADH are from ref 8.Reactions were in 33 mM Na_2_SO_4_, 0.25 mM EDTA, and 10 μM pH indicator at 25 °C under N_2_ atmosphere.

Although reaction of the ADHs with NAD^+^ with approximately saturating levels of 10 mM pyrazole essentially produces an irreversible reaction in a few seconds (Figure 1), the rate of formation of the complex depends on the concentration of pyrazole. The apparent *K*_i_ value for pyrazole was determined by steady state kinetics by competitive inhibition against oxidation of ethanol. The data (Supporting Information Table S3) provide the pH dependence, and the fitted constants are listed in Supporting Information Table S2. The pH dependence shows that some group on the enzyme–NAD^+^ complex has a p*K* value of 9.0, which is shifted to 7.8 in the enzyme–NAD–pyrazole complex. The same experiments provide pH dependencies for ethanol oxidation, and the p*K* of 8.6 for *V*_1_/*K*_m_ provides an estimate for the p*K* of the H51Q ADH–NAD^+^ complex, which was determined previously.^36^

Data at a few pH values were obtained with varied concentrations (4 or more each) for NAD^+^ and pyrazole and fitted with the equation that applies for a rapid-equilibrium or ordered mechanism (Table 2). The overall bimolecular rate constants for formation of the H51Q ADH–NAD^+^ complex (*k*_lim_/*K*_NAD_) as monitored by proton release or the increase in absorbance at 293 nm are similar to the values determined at 10 mM pyrazole, and about 20-fold smaller than those for wild-type enzyme at pH 8.^6,8,31^ Some of the progress curves could be fitted with a double exponential equation, but the two rate constants differed only by ∼ 3-fold, so that the single exponential fit was used. The NAD–pyrazole complex forms with wild-type enzyme with biphasic kinetics. These macroscopic kinetic constants are a robust, model-independent, characterization of the reaction, and the transient progress curves are suitable for model-dependent simulations.

**Table 2:** Kinetic Constants for Reaction of H51Q ADH with NAD^+^ and Pyrazole^a^

pH	Signal	*k*_lim_ (s^–1^)	*k*_lim_/*K*_NAD_ (mM^–1^ s^–1^)	*k*_lim_/*K*_Pyz_ (mM^–1^ s^–1^)	*K*_NAD_ (mM)	*K*_ia_(mM)	*K*_Pyz_ (mM)
8.0	*A*_293_	10 ± 1	16 ± 1	0.58 ± 0.02	0.59 ± 0.08	0.06 ± 0.02	17 ± 2
8.0	*A*_559_	13 ± 2	20 ± 2	0.53 ± 0.02	0.62 ± 0.12	0.05 ± 0.02	24 ± 4
9.0	*A*_293_	35 ± 2	91 ± 9	5.0 ± 0.3	0.38 ± 0.06	0.19 ± 0.05	7.0 ± 0.8
9.0	*A*_596_	40 ± 9	90 ± 20	6 ± 1	0.4 ± 0.2	0.27 ± 0.15	7 ± 3
9.4	*A*_293_	71 ± 5	144 ± 8	6.3 ± 0.3	0.49 ± 0.06	0.09 ± 0.02	11 ± 2

a At each pH, the apparent first–order rate constants were determined for reactions such as in Figure 1 with 8–10 varied concentrations of NAD^+^ (0.047–1.8 mM) and 4 concentrations of pyrazole (2, 5, 10, and 20 mM). The reaction conditions were 33 mM Na_2_SO_4_, 0.25 mM EDTA, with ∼10 μM pH indicators: phenol red, pH 8.0 (*A*_559_) or thymol blue, pH 9.0, 9.4 (*A*_596_), at 25 °C under N_2_ atmosphere. The data were best fitted with the Sequential Bi mechanism, but *K*_ia_ was not well-determined.

### Formation of H51Q ADH–NAD^+^–Trifluoroethanol Complex

Trifluoroethanol is a competitive inhibitor against varied concentrations of ethanol and uncompetitive against varied concentrations of NAD^+^, showing that it binds preferentially to the enzyme–NAD^+^ complex.^37^ For the transient studies here, the concentrations of both NAD^+^ and trifluoroethanol were varied systematically, and the reactions were followed by proton release and protein fluorescence. First order rate constants were calculated for each transient, and data for one pH are shown in Figure 3. Each set of rate constants fit best with the Ping Pong Bi mechanism (Table 3). The close correspondence of the kinetic constants for the fluorescence change and the proton release at different pH suggests that the events are coupled. In the pH ranges studied, the limiting rate constant (*k*_lim_) and the binding of NAD^+^ (*k*_lim_/*K*_NAD_) are much larger with trifluoroethanol than with pyrazole (Table 2). The kinetic constants with trifluoroethanol are similar for H51Q and wild-type ADHs (cf. Table 1 in ref 8).

**Figure 3:**
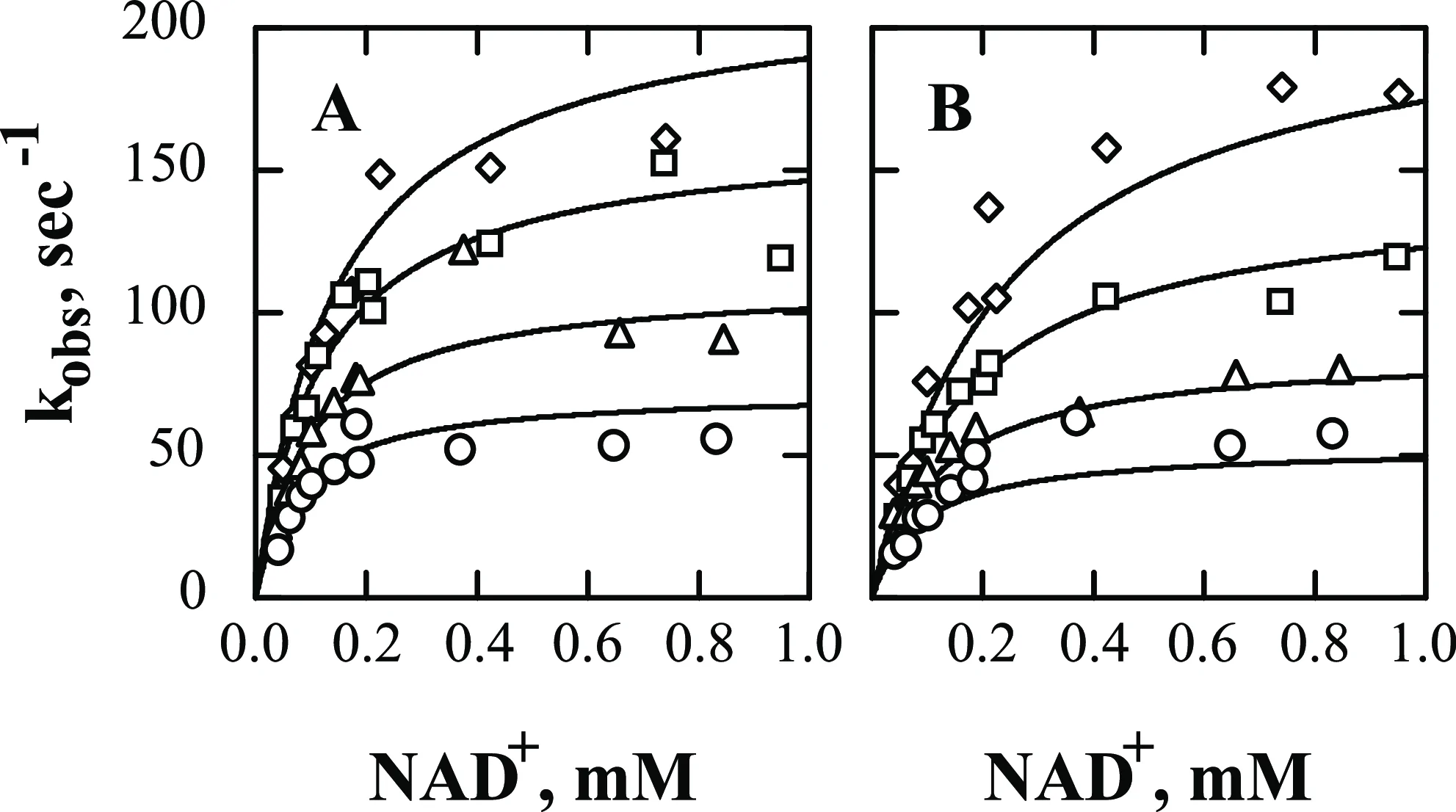
Dependence of the observed rate constants for quenching of protein fluorescence and proton release during reaction of the H51Q enzyme with NAD^+^ and trifluoroethanol. The reaction of H51Q with NAD^+^ and 4.0 (○), 7.2 (△), 14.6 (□) and 30.2 (◇) mM trifluoroethanol was monitored by (A) quenching of protein fluorescence (2 μN enzyme) and (B) by the decrease in absorbance of phenol red (5.9 μN enzyme, 10 μM phenol red, 559 nm) due to release of protons in 33 mM Na_2_SO_4_, 0.25 mM EDTA, at pH 7.0 and 25 °C under N_2_ atmosphere.

**Table 3:** Kinetic Constants for Reaction of H51Q ADH with NAD^+^ and Trifluoroethanol^a^

pH	Signal	*k*_lim_ (s^–1^)	*k*_lim_/*K*_NA_ (mM^–1^ s^–1^)	*k*_lim_/*K*_TFE_ (mM^–1^ s^–1^)	*K*_NAD_ (mM)	*K*_TFE_ (mM)
7.0	Fl	340 ± 30	1300 ± 100	20 ± 2	0.25 ± 0.04	17 ± 3
	*A*_559_	490 ± 70	1000 ± 70	14 ± 1	0.48 ± 0.09	35 ± 7
7.4	Fl	400 ± 20	1550 ± 150	21 ± 2	0.26 ± 0.05	19 ± 4
	*A*_559_	310 ± 20	1150 ± 70	15.9 ± 0.3	0.27 ± 0.03	21 ± 3
8.2	Fl	350 ± 50	1400 ± 200	19 ± 2	0.25 ± 0.06	18 ± 4
	*A*_559_	360 ± 50	1200 ± 100	12.2 ± 0.6	0.30 ± 0.06	30 ± 5
8.8	Fl	250 ± 40	1280 ± 130	22 ± 2	0.19 ± 0.04	11 ± 3

a The data were fitted with the equation for the Ping Pong Bi mechanism.^44^ The data could also be fitted with the Sequential Bi mechanism, but the estimates for *K*_ia_ were the same as the errors. This could happen if the magnitudes of *K*_ia_ are smaller than that of *K*_NAD_. Reactions used 33 mM Na_2_SO_4_ with 10 μM phenol red as a pH indicator at 559 nm.

### Stoichiometry of Proton Release

The number of protons released per enzyme subunit ([H^+^]/[E]) during reaction of the H51Q enzyme with NAD^+^ and pyrazole was determined from the transient kinetics and direct potentiometric titrations. The bell-shaped pH dependence shows that about one proton is released per enzyme subunit at pH 8 (see Supporting Information Figure S1A). The data were fitted with the equation for the mechanism in Scheme 3, where both the free enzyme and the enzyme–NAD^+^–pyrazole complex ionize in the observable pH range. The pH dependence suggests that the p*K* of ∼9.2 of some group on the free enzyme (such as the zinc-water) is shifted to a p*K* of ∼6.5 in the enzyme–NAD–pyrazole complex, where the pyrazole displaces the water and deprotonates to form the adduct observed by crystallography. The pH dependence for proton release from wild-type ADH showed only a p*K* of ∼9.5^8^, whereas a previous potentiometric study over the pH range of 6–10 showed p*K* values of 9.5 and 6.0.^25^

**Scheme 3:**
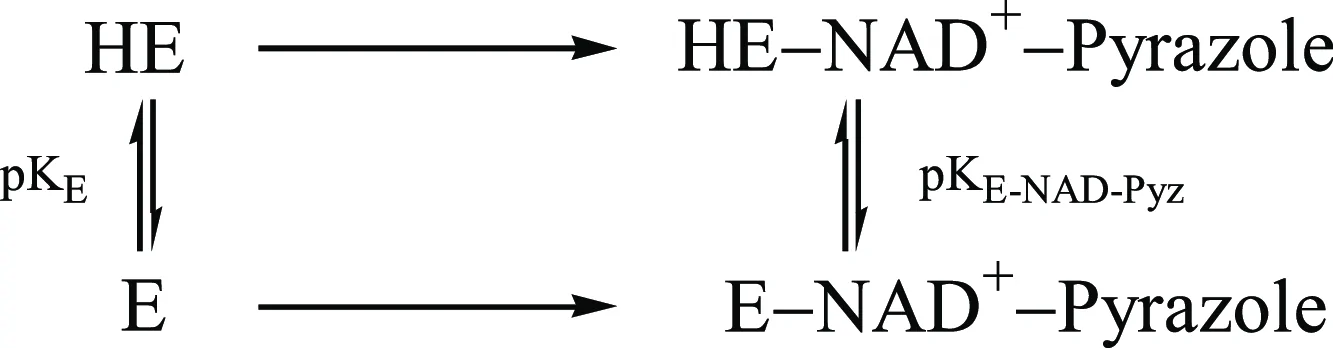
Proton Release during Formation of the Ternary Complex

One proton is released per enzyme subunit during the transient reaction of the H51Q enzyme with NAD^+^ and trifluoroethanol at pH values from 7.0 to 8.25 (Supporting Information Figure S1B). The zinc-water is replaced by the deprotonated trifluoroethanol as determined by the crystallography. The apparent p*K* values for the H51Q ADH–NAD^+^–TFE complex were not established, but any lower p*K* is less than 6. Potentiometric titrations for reaction of wild-type ADH with NAD^+^ and trifluoroethanol show that one proton is released per enzyme subunit at pH 8, and the pH dependence shows p*K* values of 6.0 and 9.5.^8,25,27^ These macroscopic studies do not determine when the proton is released in the mechanism.

### Formation of H51Q ADH–NAD^+^–Caprate Complex

Caprate is a competitive inhibitor against ethanol, binding preferentially to the wild-type ADH–NAD^+^ complex with a *K*_i_ value of 2 μM at pH 8 and a rate constant for dissociation of about 2 s^–1^. Caprate also binds to the apoenzyme with a *K*_d_ of 45 μM at pH 8 with essentially diffusion-controlled kinetics, with a rate constant >10^7^ M^–1^s^–1^.^64–67^ Caprate binds most tightly to the complex with NAD^+^ at low pH below a p*K* of 7.2, with no net release of protons in the range of pH 6.0 to 8.5.^27^ In static experiments at pH 8.5, binding of NAD^+^ releases about one proton per subunit, and binding of caprate takes up about one proton per subunit so that there is no net change. Caprate effectively traps the enzyme–NAD^+^ complex. Biphasic, fast proton release and slower uptake during reaction of the wild-type enzyme with NAD^+^ and caprate support the mechanism where the enzyme–NAD^+^ complex isomerizes and deprotonates, and then caprate replaces a hydroxide ion bound to the catalytic zinc.^8^

The binding of caprate and NAD^+^ to H51Q ADH at pH 8.0 was monitored by the change in absorbance of the pH indicator at 559 nm for proton release, or absorbance at 280 nm, or by quenching of protein fluorescence. Significantly, the biphasic, transient proton release/uptake observed with wild-type enzyme^8^ is largely (∼90%) decreased with H51Q ADH, and there is very little change in pH during the reaction at pH 8. The diminished proton release/uptake suggests that the H51Q substitution alters the p*K* of the enzyme–NAD^+^ complex or the rate constants for binding NAD^+^. X-ray crystallography with H51Q ADH shows that the carboxylate of caprate binds to the catalytic zinc close to the positively charged NAD^+^.

In contrast to the reactions with pyrazole or trifluoroethanol, the observed rate constants for the reaction with caprate depend only on concentration of NAD^+^ (Table 4). Caprate does not affect the rates of reaction, but the amplitudes of the transient progress curves for protein fluorescence and absorbance at 280 nm increase as the concentrations of caprate (0.06–0.7 mM) increase. The fast phase of the reaction with wild-type enzyme also does not depend on the concentrations of caprate, whereas the slow phase depends on concentrations of both NAD^+^ and caprate.^8^

**Table 4:** Kinetic Constants for Reaction of H51Q ADH with NAD^+^ and Caprate^a^

Signal	*k*_lim_ (s^–1^)	*k*_lim_/*K*_NAD_ (mM^–1^ s^–1^)	*K*_NAD_ (mM)
Fl	130 ± 4	520 ± 20	0.25 ± 0.02
*A*_280_	140 ± 15	410 ± 35	0.33 ± 0.06

a The observed first–order rate constants with varied concentrations of NAD^+^ and caprate were fitted with the Michaelis–Menten equation. The concentrations were varied from 0.05–1.0 mM NAD^+^ and 0.06–0.28 mM caprate for the fluorescence experiments and 0.1–0.57 mM NAD^+^ and 0.06–0.69 mM caprate for the absorbance experiments. The reactions were carried out in 33 mM Na_2_SO_4_, 0.25 mM EDTA, at pH 8.0 and 25 °C under N_2_ atmosphere.

### Kinetic Simulations of Potential Mechanisms with Varied Concentrations of Ligands

Simulations of the transient data can distinguish among possible kinetic mechanisms and provide microscopic rate constants for steps in the mechanism. For the binding of NAD^+^ with trapping of the ternary complex with pyrazole or trifluoroethanol, two different three-step mechanisms can explain the results. Previous studies showed that the initial wild-type ADH–NAD^+^ complex isomerizes and deprotonates before ligands bind to the catalytic zinc (Scheme 4).^6–8^ The isomerized enzyme is F, and the ligand—pyrazole, trifluoroethanol, caprate—binds to the zinc, replaces the zinc-bound hydroxide as the deprotonated form X. In contrast, H51Q ADH appeared (at pH 8) to isomerize much less well (with trapping by pyrazole), and the kinetic constants were strongly pH dependent.^31^ In the present study the formation of the ternary complex (*A*_293_) and proton release have similar kinetic constants (Figure 2, and Table 2), and the data can be fitted by the mechanism in Scheme 4 with rate-limiting deprotonation and isomerization of the enzyme–NAD^+^ complex (Supporting Information Table S4.1).

**Scheme 4:**
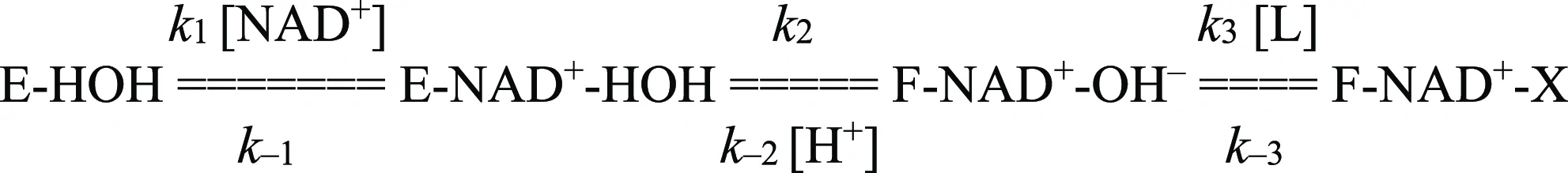
Mechanism with Deprotonation and Isomerization of the Binary Complex

However, an alternative mechanism has isomerization and deprotonation of the ternary complex (Scheme 5). The ligand to the zinc is L (protonated forms of pyrazole or trifluoroethanol that replace the water and deprotonate to form X) and F represents the isomerized form of enzyme. The structures of the wild-type and H51Q ADH complexes (8G2X.pdb, 1N92.pdb) suggest that an initial enzyme–NAD^+^–pyrazole complex could form a hydrogen bond with the hydroxyl group of Ser-48 at about 3.2 Å from N1 or N2 of pyrazole and facilitate release of a proton, which could be affected by the H51Q substitution.

**Scheme 5:**
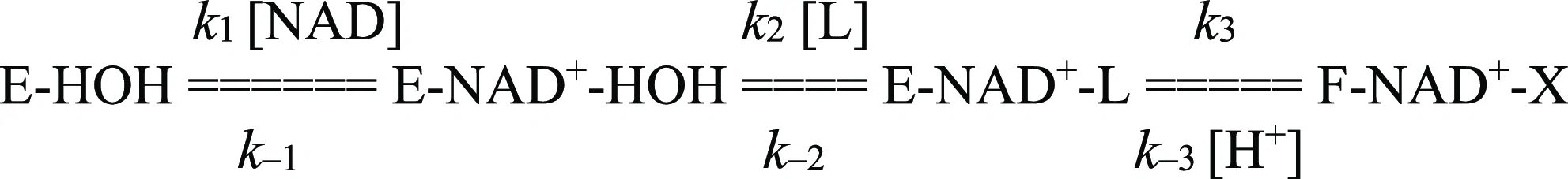
Mechanism with Deprotonation and Isomerization of the Ternary Complex

This mechanism describes the transient data just as well as the mechanism in Scheme 4 (Supporting Information Table S4.2). The simulations do not distinguish between the mechanisms, probably because the slowest unimolecular step is coupled to proton release, and the transients fit well with a single exponential rate equation and provide little information about intermediate enzyme forms with absorbance signals at 293 or 559 nm.

Transient kinetic data for binding of NAD^+^ and trifluoroethanol were simulated with Dynafit with various mechanisms (Supporting Information Table S5). Protein fluorescence appears to report on intermediate forms of enzyme (see Supporting Information). The values calculated for the dissociation constant for NAD^+^ and the *K*_d_ for trifluoroethanol from the rate constants approximate the values determined by steady state kinetics.^31^ The mechanism with isomerization of the enzyme–NAD^+^ complex and then binding of trifluoroethanol (Scheme 4, Supporting Information Table S5.2a) provides output factors for four forms of enzyme, where the fluorescence decreases in a stepwise manner, and reasonably good estimates of the rate constants for the putative isomerization (*k*_2_/*k*_–2_). The rate constants for *k*_2_ (200–400 s^–1^) are similar to those found for wild-type enzyme, whereas the rate constants for *k*_–2_ are somewhat larger than those for wild-type ADH (124 s^–1^ at pH 8^8^).

The alternative mechanism with isomerization of the enzyme–NAD^+^–trifluoroethanol complex coupled to proton release (Scheme 5) also describes the data well, and rate constants for step 3 are well estimated (Supporting Information Table S5.3). For either mechanism, the rate constants for proton release are more than 10-fold faster with trifluoroethanol than with pyrazole, perhaps as expected for an alcohol dehydrogenase.

The simulations of the binding of NAD^+^ and trifluoroethanol suggest that isomerization of the initial enzyme–NAD^+^ complex is similar for wild-type and H51Q ADHs, but establishing which step has the slow proton release for H51Q ADH is kinetically ambiguous. However, comparison of the simulations with pyrazole and trifluoroethanol suggest that the isomerization (conformational change) of H51Q–NAD^+^ complex is modestly affected as compared to wild-type enzyme (with trifluoroethanol data), and deprotonation of the ternary complex is rate-limiting with pyrazole (see later).

### Proton Transfer During Ethanol Oxidation

Study of the binding and oxidation of ethanol can inform about the kinetics of proton and hydride transfer and the conformational change. For wild-type ADH, various kinetic studies suggest that deprotonation precedes hydride transfer from alcohols whereas hydride transfer to aldehydes precedes protonation, consistent with a zinc–alkoxide as a transient intermediate.^7,27,29,30,68,69^

A preliminary experiment showed that the transient burst and steady state kinetics of wild-type enzyme in a buffer that included 33 mM Na_2_SO_4_ at pH 8 could be determined well, but the burst phase with H51Q ADH was not as well-defined as for wild-type ADH (Supporting Information Table S6). Nevertheless, the transient reactions for hydride transfer and proton release for H51Q ADH with a fixed concentration of NAD ^+^ and four varied concentrations of ethanol have essentially the same rates at each concentration of ethanol (example in Figure 4).

**Figure 4:**
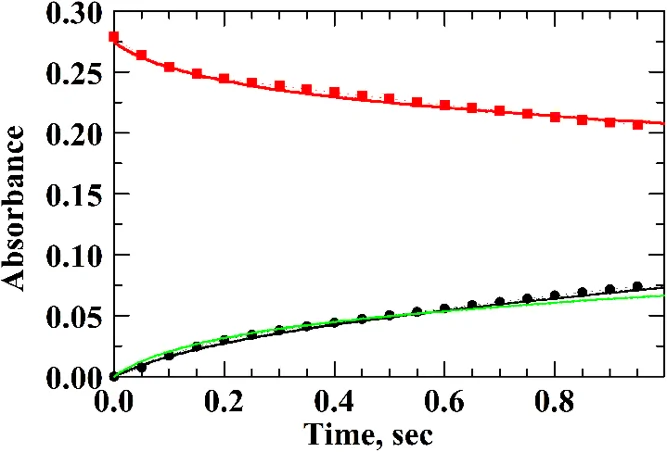
Hydrogen transfer during oxidation of ethanol. The transient reaction of 2.5 mM NAD^+^ and 6.3 mM ethanol at pH 8 and 25 °C with 8 μN H51Q ADH in a 1 cm cuvette. The lower trace shows hydride transfer to form NADH as measured at *A*_333_ nm (increasing black line, with simulation shown as black circles). The upper trace shows proton release measured at *A*_559_ nm from reaction with the basic form of phenol red (line with the data and the simulation as squares). The lower trace with the green line has the proton release data inverted, and it overlaps hydride transfer. The simulation (eight transients for hydride and proton transfer) shows that the enzyme–NAD^+^ complexes form rapidly, the alkoxide builds up as an intermediate at 0.1 s, and the steady-state phase is established at 0.3 s where 60% of the enzyme species have bound NADH.

A mechanism with proton release coupled to a postulated isomerization of the enzyme–NAD^+^ complex (EA = “FA”) fit the data well (Supporting Information Table S7). Previous simulations in phosphate buffer had no data for proton release.^36^ The mechanism with proton release coupled to formation of a postulated enzyme–NAD^+^–alkoxide complex (FAB = “FAX”) also fit the data well. For both mechanisms, estimated rate constants for unimolecular steps in the forward or reverse reactions are larger than the turnover numbers.

### X-ray Crystallography of Ternary Complexes

Structures of six ternary complexes with NAD^+^ and a ligand to the catalytic zinc were solved to examine the effects of the H51Q substitution on global and local conformations and on interactions of inhibitors in the active site. All of the crystals have the same triclinic unit cell with one dimeric molecule in the asymmetric unit with the closed conformation as found for many ternary complexes.^2^ The subunits in each dimer are almost identical and can be superposed with rmsd values of ∼0.15 Å (up to 0.2 Å) between and among the different complexes. Structures of wild-type ADH with NAD^+^ and pyrazole, trifluoroethanol, and 2,3,4,5,6-pentafluorobenzyl alcohol were determined previously at high resolution.^54,55,70^ Structures with NADH and water or *N*-cyclohexylformamide ligated to the catalytic zinc were also determined.^3,71^ The structure of the enzyme–NAD^+^–caprate complex is new for horse liver ADH1E.

The nicotinamide rings in the complexes with NAD^+^ are slightly puckered, suggesting strain in the ground state structures, whereas the ring is significantly puckered in complexes with NAD^+^ and pyrazole.^54,55,72^ The nicotinamide ring of NADH is also puckered.^3,56^

The electron density map in Figure 5 shows that positions of pyrazole and Gln-51 are clearly defined in the active site of the enzyme. Pyrazole is bound in the same position as in the complex with the wild–type enzyme.^55^ Supporting Information has close up views of the electron density map (Figure S2) and of the hydrogen bonding (Figure S3). The distance between N1 of pyrazole and the zinc is 1.6 Å and between N2 of pyrazole and C4 of nicotinamide ring is 2.1 Å (Supporting Information Table S8). The short distances are consistent with the formation of a covalent adduct and a deprotonated pyrazole in the ternary complex. The distance between the Ser-48 OG hydroxyl group and either N1 or N2 of pyrazole is about 3.2 Å in both subunits.

**Figure 5:**
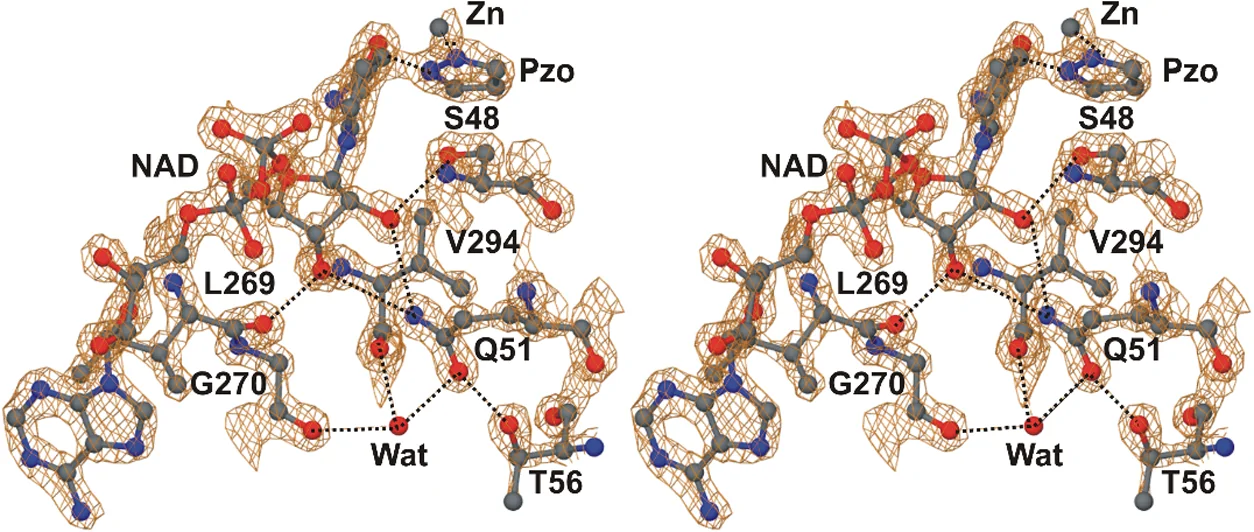
Electron density map (stereo view) defining the position of the pyrazole and Gln-51 in the H51Q ADH–NAD^+^–pyrazole complex (8G2X.pdb).

In all of the H51Q ADH structures, the major position for Gln-51 is the same, with interactions of the carboxamido-NH_2_ group with the hydroxyl groups of the nicotinamide ribose. The position of the carboxamido group of Gln-51 is well–defined, and its orientation is supported by the temperature factors of the atoms, MolProbity analysis, and hydrogen-bonding. The carboxamido NH_2_ (NE2 in the RCSB PDB) of Gln-51 interacts (at 3.2 Å) with the 2′- and 3′-hydroxyl groups of the nicotinamide ribose, and the carboxamido O (OE1) of Gln-51 is 2.6 and 2.7 Å away from the hydroxyl group of Thr-56 and a water molecule, respectively (Supporting Information Table S9). If the carboxamido group were flipped, and the -NH_2_ moiety of Gln-51 were to interact with Thr-56 and a water molecule, the 3′-hydroxyl of the nicotinamide ribose would be in close contact with two carbonyl oxygens, those of Gln-51 and Ile-269. Although the orientation of the carboxamido group of Gln-51 shown in Figure 5 is most common, other complexes show that alternative positions are possible (see below).

The H51Q substitution causes minor structural rearrangements in the active site as the interaction of Gln-51 with Thr-56 OG1 alters its association with Ser-54 OG found in wild-type ADH (Figure 6). Although glutamine and histidine residues are almost isosteric, the positions of the side chains differ in the wild-type and H51Q enzymes, and the hydrogen bonds to the nicotinamide ribose have somewhat different distances and orientations.

**Figure 6:**
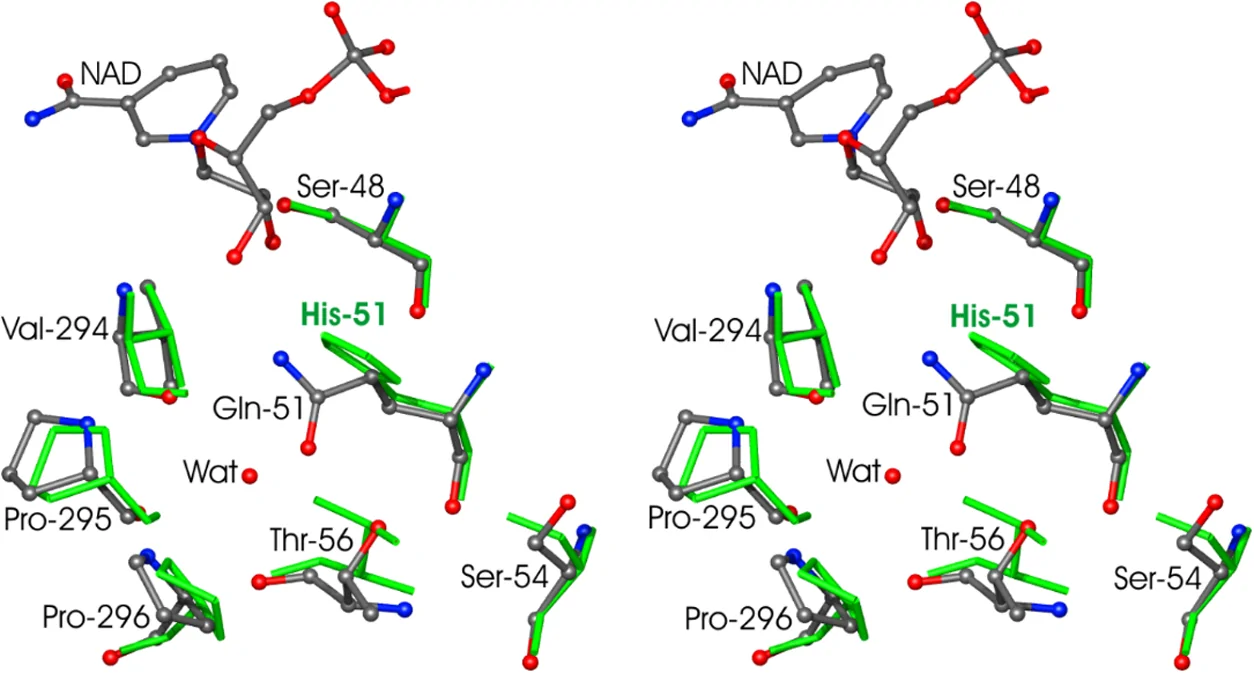
Comparison of the structures of the enzymes with His–51 and Gln-51. The coenzyme–binding domains of the H51Q ADH–NAD^+^–pyrazole (8G2X.pdb) and the wild–type ADH–NAD^+^–4-iodopyrazole (1N92.pdb) structures were superimposed with a rmsd value of 0.14 Å. The gray and color-coded ball and stick model represents the H51Q ADH complex, and the green stick model represents the wild-type ADH complex.

Electron density for the ligands in the H51Q ADH–NAD–TFE complex is shown in Figure 7. The hydroxyl oxygen of trifluoroethanol is coordinated directly to the catalytic zinc and interacts with the hydroxyl group of Ser-48. The hydrogen–bonding interactions in the active site of the complex are very similar to those for the ternary complexes with pyrazole, caprate, pentafluorobenzyl alcohol, and *N*-cyclohexylformamide, and the orientation of the carboxamido group of Gln-51 is the same, as depicted in the abstract graphic and Figure 6 and listed in Supporting Information Table S9.

**Figure 7:**
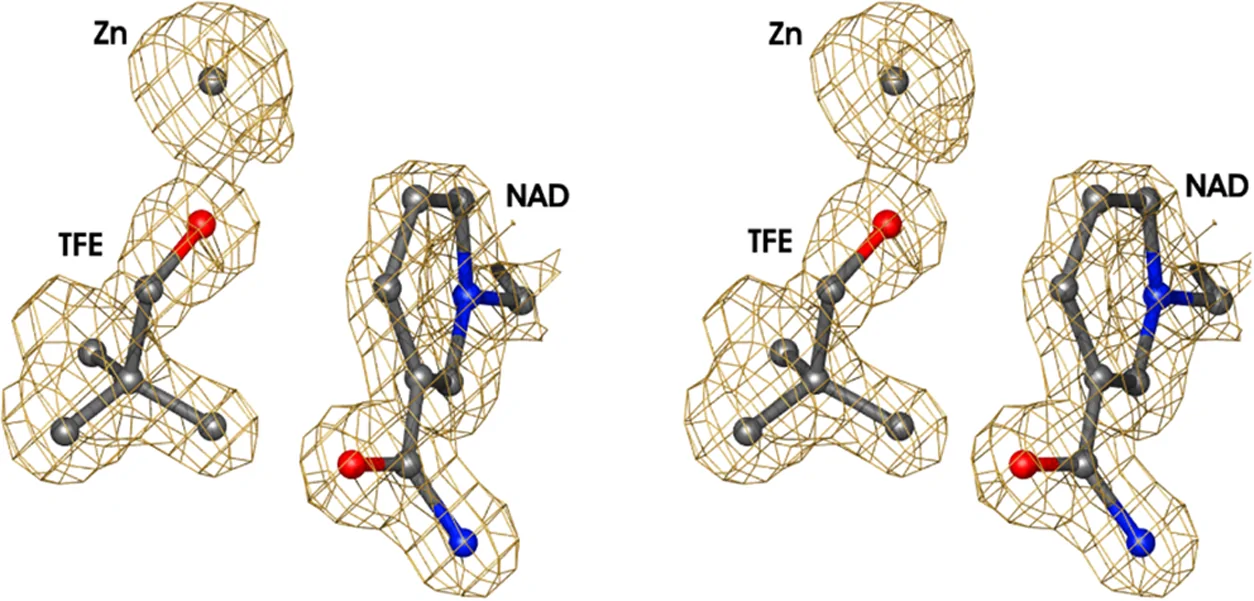
Electron density map defining the major position of 2,2,2-trifluoroethanol in the H51Q ADH–NAD–TFE complex (8G2L.pdb).

The binding of trifluoroethanol is very similar in the complexes with wild-type (4DXH.pdb) and H51Q ADHs. The distances from the oxygen of trifluoroethanol to the catalytic zinc or to the hydroxyl oxygen of Ser-48 are 2.0 and 2.5 Å, respectively (Supporting Information Table S8), and consistent with the formation of the alcoholate and a low barrier hydrogen bond.^54^ The release of one proton per subunit is also consistent with deprotonation of the alcohol (Supporting Information Figure 1B). The distance between the methylene carbon of trifluoroethanol and C4N of NAD (3.4 Å) suggests that the complex resembles a proposed “tunneling ready state” for hydride transfer.^73^ On closer inspection of the electron density maps, an alternative position (∼40% occupancy) is found for the trifluoromethyl group, which appears to be “wagging” a little and can reflect local dynamics. Such flexibility in binding was also illustrated in F93W ADH.^72^ (The alternative positions are best viewed by downloading the structural models.)

The structure of the H51Q enzyme–NAD^+^–caprate complex has one oxygen of the carboxylate group coordinated directly to the catalytic zinc and a hydrogen bond with Ser-48 OG (Figure 8). The structure also shows that the amido group of Gln-51 hydrogen bonds to the 2′-hydroxyl group of the nicotinamide ribose, which interacts with Ser-48, which in turn donates a hydrogen bond to the oxygen of the ligand bound the catalytic zinc, forming the proton relay system typical of structures with NAD^+^ and an alcohol (Supporting Information Table S8).

**Figure 8:**
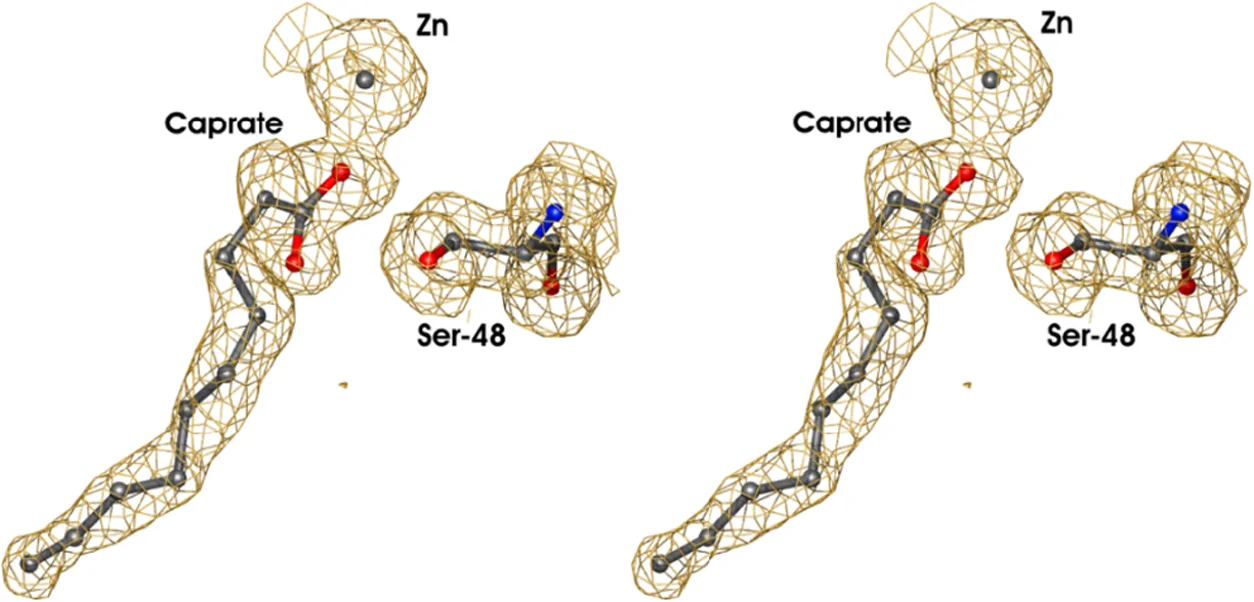
Electron density map showing the major position of caprate in the complex with H51Q ADH–NAD^+^ (8G2S.pdb).

Caprate has been used in equilibrium binding and displacement studies of ligands bound to the catalytic zinc in complexes with NAD^+^.^40,65,66^ However, structural information about coordination of carboxylates in the active site of ADH is rather scarce and contradictory. In one structure, the carboxyl group of cholic acid is ligated to the catalytic zinc in the ternary complex of horse ADH1S, an isoenzyme active on steroids, with substitutions of six amino acid residues relative to ADH1E.^37,74^ In contrast, X–ray data for glutathione-dependent formaldehyde dehydrogenase complexed with NAD^+^ and either dodecanoate or 12-hydroxydodecanoate were interpreted in terms of a model where the carboxylate moiety of the inhibitors is coordinated to a remote anion binding site, whereas the methyl or hydroxymethyl end of the inhibitors approaches the catalytic zinc.^75^ The structure of the tetrameric ADH from Aeropyrum pernix solved at 1.62 Å complexed with NADH was discovered to have the carboxyl group of endogenous octanoate bound to the catalytic zinc and strangely stabilized by a short interaction of one oxygen with a carboxylate of Asp-168, which is also ligated to the catalytic zinc.^76^ Human Class 2 (π) ADH was crystallized in the presence of 0.2 M ammonium acetate, and a carboxylate oxygen of acetate is ligated to the catalytic zinc and linked in a hydrogen bonded system to Thr-44 OG1, NAD O2D, water, and Thr-52 OG1 to solvent (3COS.pdb).

The electron density map defines a good position for caprate in the active site of the H51Q enzyme (Figure 8). However, close examination of the maps shows that there is an alternative binding mode with about 33% occupancy where the carboxylate group is rotated with one oxygen still bound to the zinc and the aliphatic side chain fitting in the electron density, with no close contacts with protein residues in the substrate binding channel. However, the second oxygen of the carboxylate in the “A” conformation closely interacts with the nicotinamide ring, whereas in the “B” conformation it interacts with His-67 and Phe-93. Gln-51 is bound in the same position as in the complexes with pyrazole, trifluoroethanol, and *N*-cyclohexylformamide. Modeling of the structure with AlphaFold3 ^77^ found a position for the carboxylate similar to the alternative position suggested by the X-ray data, and Gln-51 in the alternative position (overlapping with the position where His-51 is found in wild-type ADH) identified in the high resolution structure with NAD^+^ and pentafluorobenzyl alcohol (8G4V.pdb). Wild-type ADH can slowly oxidize aldehydes (presumably as the hemiacetals) to carboxylates, and the present complex with NAD^+^ and caprate then represents an abortive substrate-product complex.^78–81^ It appears that caprate can bind in different positions, which reflect the local structural mobility that can accommodate a hemiacetal or secondary alcohol as substrates.

A high resolution structure of the complex with NADH and *N*-cyclohexylformamide provides a model for complexes with NADH and aldehydes. The structure with H51Q ADH is very similar to those of wild-type ADH with NADH and *N*-cyclohexylformamide or *N*-benzylformamide or *N*-1-methylhexylformamide.^3,71^ In particular, the distances between the oxygen of the formamide and the catalytic zinc or the hydroxyl group of Ser-48 are 0.1–0.2 Å longer than in the complexes with the fluoroalcohols, consistent with a carbonyl oxygen (Supporting Information Table S8). The nicotinamide ring is also puckered with bond distances typical for NADH.^3,54^ Gln-51 is in the same position as found for the other complexes of H51Q ADH, which suggests that the hydrogen bonding in the proton relay system is similar, although the carbonyl oxygen bound to the catalytic zinc is not charged, as compared to the alkoxide in complexes with alcohols.

A structure with NAD^+^ and 2,3,4,5,6-pentafluorobenzyl alcohol was determined at atomic resolution (Table 1) and is illustrated in the graphical abstract. The alcohol is bound in the same location as in wild-type ADH (4DWV.pdb), and the predominant position for Gln-51 (“A”, 70%) is the same as in the other five structures determined in this work. However, the electron density supports an alternative position (“B”) with an occupancy of about 30% in each subunit. This position almost retains the interactions of the carboxamido NE2, but flips the position for OE1, which then interacts closely with the nicotinamide ribose 2′-hydroxyl group (Supporting Information Table S9). The alternative conformations show that the position of Gln-51 is flexible, which is relevant for the dynamics of the enzyme reaction, where proton transfer between the substrate bound to the zinc and bulk solvent might require flipped orientations of Gln-51 or His-51. The carboxamido group in the alternative conformation for Gln-51 overlaps the position of the imidazole group of His-51 observed in many complexes with wild-type ADH, such as 4DWV.pdb and 4DXH.pdb.^54^

The H51Q enzyme complexed with NADH and water ligated to the zinc also has alternative positions for Gln-51, with occupancies of about 50:50. Although this complex was initially produced with 2 mM NAD^+^ and 1 mM trifluoroethanol, the NAD^+^ was apparently reduced by oxidation of the crystallization agent 2,4-methylpentanediol because the low concentration of inhibitor did not prevent formation of NADH. Wild-type ADH can form abortive complexes with NADH and benzyl alcohols during the crystallization with NAD^+^ and the alcohols.^56^

The alternative position for Gln-51 in the H51Q ADHs is similar to the major position found in the doubly mutated H51Q/K228R ADH crystallized with NAD^+^ and difluorobenzylalcohols.^36^ Rerefinement of the structures reported as 1QV6.pdb and 1QV7.pdb, confirmed the alternative conformations, but show that they are abortive NADH–alcohol complexes (Supporting Information Table S10). The critical evidence is that the zinc–alcohol O1 distance is 2.2 Å and Ser-48 OG to O1 is ∼2.7 Å, which are consistent with a protonated alcohol bound to the zinc, in contrast to 2.0 and 2.5 Å in the atomic resolutions structures with NAD^+^ and pentafluorobenzyl alcohol (4DWV.pdb and 8G4V.pdb) or trifluoroethanol (4DXH.pdb).^54^ Moreover, the distance between the methylene carbon of the benzyl alcohols to C4N of the nicotinamide ring (C7 to NAD C4N) is ∼4 Å in complexes with NADH, consistent with a rotation of the alcohol into a “nonproductive” mode of binding, as compared to 3.4 Å in complexes with NAD^+^.^34,56^ The hydroxyl group of an alcohol probably donates a hydrogen to Ser-48 OG, which donates a hydrogen bond to the nicotinamide ribose O2D, which donates a hydrogen bond to the Gln-51 carboxamido OE1 (see Figure 6 in ref 36). The hydrogen bonding is inferred from the structures, but these structures suggest that the H51Q substitution produces an enzyme that is sensitive to the state of ionization of ligands bound to the catalytic zinc and dynamically responds to catalytic events. In the wild-type enzyme, His-51 can participate in the proton transfers (Scheme 2), and the imidazole group might move when a proton is released to solvent, but only one major position is observed in ternary complexes with NAD^+^ and alcohols or with NADH and formamides or sulfoxides, which mimic reactive substrates.^3,82,83^

X–ray crystallography demonstrates that the ternary complexes of both the wild-type and H51Q ADHs have essentially the same holo–enzyme conformations, and either residue at position 51 (histidine or glutamine) participates in hydrogen–bonding interactions with the nicotinamide ribose in the active site. Furthermore, the H51Q substitution has no obvious effects on the binding modes of the coenzyme and inhibitors or substrate analogs although binding constants and pH dependencies are altered.

## Discussion and Conclusions

### The H51Q Substitution Affects Formation of Ternary Complexes

Previous kinetic studies showed that the H51Q substitution raised p*K* values characterizing several kinetic constants for the enzyme mechanism and decreased catalytic activity at pH 8.^36^ Here, two different signals show that the H51Q substitution decreases the kinetic constants for formation and deprotonation of the ternary complexes (*k*_lim_/*K*_L_) with pyrazole and trifluoroethanol (Table 5). The three kinetic constants determined with the signal at *A*_293_ for the formation of the enzyme–NAD–pyrazole adduct and at *A*_559_ for proton release, decrease by 10–300-fold as compared with wild-type ADH. However, the kinetic constant for NAD^+^ binding (*k*_lim_/*K*_NAD_) during formation of the ternary complex with trifluoroethanol, as determined with proton release and protein fluorescence, which reports on the binding of NAD^+^ and subsequent conformational changes, are only modestly altered (<2-fold). As monitored by the *A*_280_ signal and fluorescence in the presence of caprate, NAD^+^ binds with about the same kinetic constants to both wild-type and H51Q ADHs.

**Table 5:** Comparisons of Macroscopic Kinetic Constants for Formation of Ternary Complexes^a^

Ligand, L	ADH	Signals	*k*_lim_ ^b^ (s^–1^)	*k*_lim_/*K*_NAD_ (mM^–1^ s^–1^)	*k*_lim_/*K*_L_ (mM^–1^ s^–1^)
Pyrazole	Wild-type	*A*_293,_ *A*_559,_ Fl	160	410	210
	H51Q	*A*_293,_ *A*_559_	12	18	0.55
Trifluoroethanol	Wild-type	*A*_559,_ Fl	1100	640	270
	H51Q	*A*_559,_ Fl	370	1300	18
Caprate	Wild-type	*A*_280,_ Fl	310	270	1000
	H51Q	*A*_280_, Fl	130	460	--^c^

a Data at pH 8 for wild-type ADH from Table 1 in ref 8 and for H51Q ADH from Tables 2, 3, and 4 are compared. The signals are *A*_293_ for formation of the adduct of NAD^+^ and pyrazole, *A*_559_ for proton release, Fl for quenching of protein fluorescence, *A*_280_ for binding of the adenine moiety of NAD^+^ and caprate.

b The maximum limiting rate constant at saturating levels of NAD^+^ and ligand.

c Independent of caprate concentration.

Simulations of two different mechanisms for the formation of the ternary complexes with pyrazole, trifluoroethanol, and ethanol fit the data well (Table 6). The mechanism with the proton release during the HEA = FA isomerization (Scheme 4, FA columns on the left) cannot be an overall general explanation because the isomerization occurs before the ligand traps the FA species, and the rate constants for step 2 should be about the same, being independent of the ligand. In contrast, the mechanism with the EAL = FAX deprotonation (Scheme 5, FAX columns on the right) is better because the rate constants for binding of the ligands and acid dissociation constants in the ternary complexes differ, as expected. The p*K* values for EAL species increase about 1.5 unit for H51Q ADH as compared to those for complexes with the wild-type enzyme in other studies.^42,66,84,85^ The p*K* values for deprotonation of the NAD^+^–pyrazole or trifluoroethanol complexes are similar (5.7 or 5.8), lowered from the pKs of free pyrazole (11.5) and trifluoroethanol (12.4).

**Table 6:** Comparison of the Simulations for Ternary Complex Formation with Proton Release during Isomerization of E–NAD^+^ (Step 2) or from the E–NAD^+^–Ligand Complex (Step 4)^a^

Step	1	2	3	1	3	4
Mechanism	HE = HEA = FA = FAX			HE = HEA = EAL = FAX		
Reaction	FA, PYZ^b^	FA, TFE^c^	FA, ETOH^d^	FAX, PYZ^b^	FAX, TFE^c^	FAX, ETOH^d^
*k*_1_ (μM^–1^ s^–1^)	0.051 ± 0.001	1.76 ± 0.08	2.8	0.029 ± 0.001	1.68 ± 0.02	2.8
*k*_–1_ (s^–1^)	3.5 ± 0.1	120 ± 20	200	2.33 ± 0.06	142 ± 4	200
*k*_2_ (s^–1^)	**3.64 ± 0.011**	**231 ± 2**	**11.0** ± **0.1**	NR	NR	NR
*k*_–2_ (s^–1^)	**3900 ± 2000**	**600 ± 10**	**2200** ± **600**	NR	NR	NR
*k*_3_ (μM^–1^ s^–1^)	0.7 ± 0.4	0.065 ± 0.002	18 ± 4	0.6 ± 0.4	0.032 ± 0.001	0.27 ± 0.09
*k*_–3_ (s^–1^)	0.02	5.4 ± 0.1	154 ± 5	3900 ± 2600	190 ± 10	480 ± 160
*k*_4_ (s^–1^)	NR	NR	NR	**3.63 ± 0.04**	**162 ± 2**	**810 ± 550**
*k*_–4_ (s^–1^)	NR	NR	NR	**0.019 ± 0.0004**	**4.2 ± 0.08**	**970 ± 660**
p*K*	11	7.8	10.3	5.7	5.8	8.1

a Compiled from Supporting Information Tables S4, S5, and S7. Symbols: H, on water bound to catalytic zinc; E open conformation, F closed conformation; A, NAD^+^; L, protonated ligand (pyrazole, trifluoroethanol, or ethanol); X, deprotonated ligand. Bold font for proton release; italic font for fixed rate constants. The estimated rate constants for binding of NAD^+^ for the simulations with pyrazole or trifluoroethanol are highly correlated (as for a rapid equilibrium step), but the calculated dissociation constants are about the same (70–90 μM). The rate constant for the reverse protonation step was converted to first order by multiplying by the hydronium concentration, but the p*K* is calculated from the bimolecular constant at the pH of the reaction. NR, “not relevant”. The same transient data for each ligand were used for the two different mechanisms.

b Pyrazole binding with data for proton release, pH 8.

c Trifluoroethanol binding at pH 7.4 with data for protein fluorescence and proton release.

d Ethanol oxidation, NADH formation and proton release, at pH 8, but the concentration of NAD^+^ was not varied, and the rate constants for step 1 were estimated in other studies and fixed.

### The H51Q Substitution Modestly Alters Isomerization of the Enzyme–NAD^+^ Complex

Previous transient kinetic data on formation of ternary complexes of wild-type ADH were explained by a mechanism in which the initial enzyme–NAD^+^ complex isomerizes, coupled with ionization of the zinc-water with a p*K* of 7.3, followed by binding of caprate, pyrazole or trifluoroethanol to the deprotonated complex (see Scheme 4 or Table 3 in ref 8). It was suggested that proton release might be facilitated by His-51, but this needed to be confirmed.^8,31^

The kinetics of the fluorescence changes and the structural studies suggest that both the wild-type and H51Q ADHs isomerize and change conformation after binding NAD^+^ (EA = FA). The macroscopic kinetic constants for the binding of NAD^+^ (*k*_lim_/*K*_NAD_, Table 5) in the presence of trifluoroethanol or caprate to wild-type and H51Q ADHs are about the same (within 2-fold). The microscopic first order rate constants estimated by simulation of the isomerization of the enzyme–NAD^+^ complex with trapping by trifluoroethanol (*k*_2_/*k*_–2_) change from 550/120 for wild-type ADH to 290/190 for H51Q ADH in 33 mM Na_2_SO_4_ at about pH 8 (Table 3 in ref 8 and Supporting Information Table S5.2).

The apparent equilibrium dissociation constant for NAD^+^ with H51Q ADH (*K*_NAD_, Supporting Information Table S1) is essentially pH independent, in contrast to the ∼30-fold tighter binding at high pH compared to low pH for wild-type ADH (Supporting Information Table S11).^25^ Release of about one proton per subunit during the formation of the ternary H51Q ADH–NAD^+^–pyrazole complex suggests that the apoenzyme has a p*K* of ∼9.2 (Supporting Information Figure 1A), similar to the p*K* of ∼9.5 for wild-type enzyme, which was assigned to the zinc–water.^19^

The p*K* value for the enzyme–NAD^+^–water complex can be estimated from the pH dependences for the steady-state inhibition of ethanol oxidation by trifluoroethanol or pyrazole, which are 8.5 and 9.0 for H51Q ADH (Supporting Information Table S11). In contrast, the p*K* value for the wild-type enzyme–NAD^+^–water complex is ∼7.3, as estimated from the kinetics of transient proton release, pH dependence of equilibrium binding, and rate constants for dissociation of the complex.^8,11,22^ It appears that the p*K* for the H51Q ADH–NAD^+^–zinc–water complex is about 1.5 unit higher than in wild-type ADH, and the isomerization toward the closed conformation, which could be stabilized by the zinc-hydroxide, is not facilitated as much when His-51 in wild-type ADH is substituted. The increase of the p*K* is consistent with the diminished proton release when NAD^+^ binds in the presence of caprate. The ionizing group in H51Q ADH is probably the zinc-water (see Supporting Information).

The H51Q substitution modestly affects the rate constants for the isomerization (conformational change), rather than greatly favoring the open form as suggested previously.^31^ When binding of NAD^+^ to H51Q ADH is monitored by formation of the complex with pyrazole (Δ*A*_293_), deprotonation of the ternary complex is the rate-limiting step, and isomerization of the enzyme–NAD^+^ complex is not a kinetically visible step. Steady-state kinetics of horse liver and yeast ADHs also indicate isomerization of ADH–NAD^+^ complexes (Supporting Information).

### His-51 Facilitates Deprotonation of Enzyme–NAD^+^ Complexes

The pH dependencies for binding of inhibitors can provide the p*K* values of the water and other ligands bound to the catalytic zinc in apoenzyme and in complexes with coenzymes. The H51Q substitution raises the p*K* values of the enzyme–NAD^+^ complexes by about 1.4 units (Table 7). His-51 appears to act as a base to lower the p*K* values of the zinc-ligands, pyrazole, trifluoroethanol, and ethanol, in the ternary complexes. The pH dependencies for binding suggest that the ligands bind faster when the enzyme–NAD^+^–zinc–water is deprotonated. The p*K* values of water in the apoenzyme and the enzyme-NADH complex are not altered by the H51Q substitution although the proton relay system is present (1YE3.pdb^3^ and 8G41.pdb) because His-51 is probably deprotonated above pH 8. As with wild-type ADH, the p*K* of the zinc-water in the apoenzyme decreases in the complex with NAD^+^ and increases in the complex with NADH.^11,86^ The altered p*K* values for the H51Q ADH complexes with NAD^+^ and water or ethanol significantly affect the catalytic reactions (Supporting Information Table S11).^36^

**Table 7:** p*K* Values Assigned to Enzyme Forms of Wild-Type and H51Q ADHs^a^

Enzyme Complex	Wild-type^b^	H51Q^c^
Ligands	p*K* values	p*K* values
Water	9.2, 9.5, 9.6	9.2, 9.5
NAD^+^, water	6.7, 7.2, 7.3, 7.6	8.4, 8.5, 8.6, 9.0
NAD^+^, pyrazole	<4, 4.7	5.7, 6.5
NAD^+^, trifluoroethanol	4.3, 4.6	5.8, 5.8
NAD^+^, ethanol	6.4	7.8, 8.1
NADH, water	10.5, 11.2	10.7

a The p*K* values were estimated from various experiments, which give a range of values: binding of NAD^+^ or NADH; steady-state, inhibition, and transient kinetics; and simulations.^8,36^

b Key references 11,25,27,66,84,87 .

c The p*K* values are collected from Table 6 and Supporting Information Figure S1 and Tables S4.2, S5.3b, and S11.

### His-51 or General Bases Contribute to Catalysis

The H51Q substitution shifts the pH dependencies and modestly changes catalytic efficiencies and turnover numbers for the forward and reverse reactions with ethanol and acetaldehyde at pH 8. The data for horse ADH were discussed previously^36^ and are summarized in Supporting Information Table S11. Gln-51 would eliminate base catalysis by His-51, and it might hinder proton transfers via the hydrogen-bonded system. Other substitutions of His-51 have different effects, as found with the substitutions of the homologous His-48 in yeast ADH with serine, glutamate, or glutamine residues.^88,89^

When His-51 is substituted, proton transfer in the enzyme–NAD^+^–alcohol complex can be catalyzed by general bases. The H51Q substitution in human ADH1B1 decreases catalytic efficiency (*V*_1_/*E*_t_ *K*_b_) for ethanol oxidation by 6-fold at pH 7, but activity is restored by general base catalysis with HPO_4_^2–^, glycylglycine, glycine, and hydroxide, as indicated by a linear Brønsted relationship with a slope of 0.53.^90^ Activity of H51Q ADH and the homologous yeast (*Saccharomyces cerevisiae*) ADHs with substitutions of His-48 exhibit unusual pH dependencies for ethanol oxidation, and base catalysis might be involved in proton transfer.

The H48Q substitution in yeast ADH decreases catalytic efficiencies on ethanol and acetaldehyde by ∼10-fold at pH 7.3, and the H48S substitution decreases the efficiencies by 3-fold.^88,89^ The H48Q and H48S ADHs show linear pH dependencies (slopes 0.32–0.47) for log *V*_1_/*E*_t_ *K*_b_ and log *V*_1_/*E*_t_ versus pH for oxidation of ethanol over the pH range from 5.5 to 9.2. This was attributed to base catalysis by hydroxide with a low activity coefficient in the protein environment.^88,89^ Deuterium isotope effects show that hydride transfer is a major rate-limiting step. In contrast, the pH dependence of *V*_1_/*E*_t_ for doubly substituted H44R/H48Q yeast ADH is described by p*K* values of 7.0 and 9.2, similar to the p*K* values of 7.6 and 9.3 for horse H51Q ADH.^91^ The “wavy” pH dependences can result from “sticky” substrates and different effects of pH on partly rate-limiting steps.^92^ Arg-44 in yeast ADH, or Arg-47 in horse interact with the pyrophosphate of the coenzyme, and might modulate specific base catalysis by hydroxide, whereas His-44 in yeast ADH allows hydroxide to access the 2′-hydroxyl group of NAD^+^.

## Supplementary Material



## Data Availability

The data underlying this study are available in this article and the Supporting Information. Transient kinetic data and scripts for simulations are available on request.

## Notes

X-ray crystallography methods. Apparent kinetic constants for transient formation of H51Q ADH–NAD–pyrazole complexes at varied pH (Table S1); pH Dependences for reactions of H51Q ADH with NAD^+^ and pyrazole (Table S2); pH Dependence for inhibition by pyrazole of ethanol oxidation by H51Q ADH (Table S3). Stoichiometry of proton release (Figure S1); microscopic rate constants for reaction of H51Q ADH with varied concentrations of NAD^+^ and pyrazole for either isomerization of the enzyme–NAD^+^ complex or the enzyme–NAD^+^–pyrazole complex (Table S4); microscopic rate constants for reaction of H51Q ADH with varied concentrations of NAD^+^ and trifluoroethanol for either isomerization of the enzyme–NAD^+^ complex or the enzyme–NAD^+^–trifluoroethanol complex (Table S5); linking the binding of NAD^+^ to quenching of protein fluorescence and the conformational change. Kinetic constants for oxidation of ethanol at pH 8 (Table S6); simulations of the catalytic reaction with isomerization of the enzyme–NAD^+^ complex or the enzyme–NAD^+^–alcohol complex (Table S7); electron density maps for the positions of pyrazole and Gln-51 (Figure S2); hydrogen-bonding interactions in the active site of the H51Q ADH–NAD–pyrazole complex (Figure S3); interactions for hydride and proton transfer in various complexes of wild-type and H51Q ADHs (Table S8); interactions of Gln-51 in active sites of complexes of H51Q ADH (Table S9); alternative interactions of benzyl alcohols in active sites of complexes of ADH (Table S10); ionization of the zinc ligands are most relevant for pH dependencies of H51Q ADH. pH Dependences of kinetic constants for forward and reverse reactions of horse wild-type and H51Q ADHs (Table S11); steady-state kinetics of horse liver and yeast ADHs also indicate isomerization of ADH–NAD^+^ complexes. Steady-state kinetic constants for H51Q/K228R ADH at varied pH (Table S12); review of kinetic data in on the effects of the H51Q substitution on catalytic activity. General importance of the proton relay system. Future investigations (PDF)

## Abbreviations

ADH: liver alcohol dehydrogenase
H51Q: His-51 to Gln substitution (same style for substitutions of other amino acids)
MPD: 2-methyl-2,4-pentanediol
Pyz or Pzo: pyrazole
PFB: 2,3,4,5,6-pentafluorobenzyl alcohol
TFE: 2,2,2-trifluoroethanol, CXF, *N*-cyclohexylformamide.
