# Supplementary material for: Histidine-51 Facilitates the Conformational Change and Deprotonation of Horse Liver Alcohol Dehydrogenase–NAD+ Complexes
Source: Biochemistry. 2026 Oct 22;65(21):3486–500. doi: 10.1021/acs.biochem.6c00532 (PMC13738346; doi:10.1021/acs.biochem.6c00532)
Supplement: Supplementary file 1 [file bi6c00532_si_001.pdf]

SUPPORTING INFORMATION

Histidine-51 Facilitates the Conformational Change and Deprotonation of Horse Liver Alcohol Dehydrogenase–NAD^+ Complexes

Bryce V. Plapp*

Department of Biochemistry and Molecular Biology, The University of Iowa, Iowa City, Iowa 52246 USA

* corresponding author: bv-plapp@uiowa.edu

TABLE OF CONTENTS

X-ray crystallography methods S2
Table S1: Apparent kinetic constants for transient formation of H51Q ADH–NAD–pyrazole complexes at varied pH S3
Table S2: pH Dependences for reactions of H51Q ADH with NAD^+ and pyrazole. S4
Table S3: pH Dependence for inhibition by pyrazole of ethanol oxidation by H51Q ADH S4
Figure S1: Stoichiometry of proton release S5
Table S4: Microscopic rate constants for reaction of H51Q ADH with varied concentrations of NAD^+ and pyrazole for either isomerization of the enzyme–NAD^+ complex or the enzyme–NAD^+–pyrazole complex S7
Table S5: Microscopic rate constants for reaction of H51Q ADH with varied concentrations of NAD^+ and trifluoroethanol for either isomerization of the enzyme–NAD^+ complex or the enzyme–NAD^+–trifluoroethanol complex S8
Linking the binding of NAD^+ to quenching of protein fluorescence and the conformational change S9
Table S6: Kinetic constants for oxidation of ethanol at pH 8 S9
Table S7: Simulations of the catalytic reaction with isomerization of the enzyme–NAD^+ complex or the enzyme–NAD^+–alcohol complex S10
Figure S2: Electron density maps for the positions of pyrazole and Gln-51 S11
Figure S3: Hydrogen-bonding interactions in the active site of the H51Q ADH–NAD–pyrazole complex S11
Table S8: Interactions for hydride and proton transfer in various complexes of wild-type and H51Q ADHs S12
Table S9: Interactions of Gln-51 in active sites of complexes of H51Q ADH S12
Table S10: Alternative interactions of benzyl alcohols in active sites of complexes of ADH S13
Ionization of the zinc ligands are most relevant for pH dependencies of H51Q ADH S13
Table S11: pH Dependences of kinetic constants for forward and reverse reactions of horse wild-type and H51Q ADHs S14
Steady-state kinetics of horse liver and yeast ADHs also indicate isomerization of ADH–NAD^+ complexes S15
Table S12: Steady-state kinetic constants for H51Q/K228R ADH at varied pH S16
Review of kinetic data on the effects of the H51Q substitution on catalytic activity S16
General importance of the proton relay system S18
Future investigations S18
References S19

X-ray Crystallography Methods. The H51Q enzyme was dialyzed extensively against filter-sterilized 50 mM ammonium *N*-[tris(hydroxymethyl)methyl]-2-aminoethanesulfonate buffer, 0.25 mM EDTA, pH 7.0 at 4 °C (pH 6.7 at 25 °C) before about 1 ml of 10 mg/ml protein was placed in a washed dialysis bag and put into 10 ml of the same buffer in a small bottle. Inhibitors were dissolved before adding coenzyme to the bottle, and then the concentration of 2-methyl-2,4-pentanediol (MPD, purified by treatment with charcoal) was raised to 10%, followed by 1% daily increases until crystals formed at about 13% MPD, and then with 0.2% increases as crystals grew. Over several more days, the concentration of MPD was finally raised to 25%, which serves as the cryoprotectant for X-ray crystallography at 100 K. The crystals of the NAD^+–pyrazole complex were formed with 2 mM NAD^+ and 15 mM pyrazole (8G2X.pdb). Crystals also formed with 2 mM NAD^+ and 15 mM 2,3,4,5,6-pentafluorobenzyl alcohol (8G4V.pdb). Crystals (8G39.pdb) formed with 1 mM NADH and 15 mM *N*-cyclohexylformamide (K_i = 0.18 mM at pH 7[1]). Crystals formed with 2 mM NAD^+ and 1 mM sodium caprate, but the X-ray data showed low occupancy for caprate and perhaps some NADH. Some of these crystals were allowed to react with 1 mM acetaldehyde (70 min at 4 ºC) to oxidize NADH that formed due to oxidation of endogenous alcohols (probably MPD), followed by a soak with 100 mM sodium caprate in mother liquor solution (2 h) prior to data collection (8G2S.pdb). Crystals formed with 2 mM NAD^+ and 0.1 mM 2,2,2–trifluoroethanol at 13% MPD, but because the concentration of trifluoroethanol was relatively low (K_i = 0.16 mM[1]), X-ray data showed that the crystals had NADH and a water ligated to the zinc (8G41.pdb); some of these crystals were soaked (2 weeks) in the crystallization medium with 25% MPD and 100 mM trifluoroethanol prior to data collection to produce the complex with NAD(H) and trifluoroethanol (8G2L.pdb).

The crystals were flash vitrified in liquid nitrogen, and X-ray data were collected at 100 K with an R-Axis IV^{++} with a rotating anode generator at a wavelength of 1.5418 Å, typically with 0.5º oscillations for 180º or 360º total rotation with a crystal to detector distance of 70 or 80 mm and an exposure time of 3–5 min. The images were integrated with d*TREK and scaled with version 9.9.9.8L.[2] The data for the NAD^+–pyrazole complex (8G2X.pdb) were collected at 70 mm distance with 0.5º oscillations for 180º total rotation. Data for the complex with NAD^+ and 0.1 mM trifluoroethanol (8G41.pdb) were collected with 0.5º oscillations for 360º total rotation and 3 min exposures at 80 mm distance. Data for two crystals soaked with 0.1 M trifluoroethanol (8G2L.pdb) were collected with 0.5º oscillations for 360º total rotation for each crystal and merged during processing. Data for the crystals with NAD^+ and 100 mM sodium caprate (8G2S.pdb) were collected at 80 mm distance, 0.5º oscillation for 360 total rotation and 3 min exposure. Data for the crystal with NADH and *N*-cyclohexylformamide (8G39.pdb) were collected at 70 mm distance, with 5 min exposures, 0.3º oscillations over 220º total. High resolution data for the crystal with NAD^+ and 2,3,4,5,6-pentafluorobenzyl alcohol (8G4V.pdb) were collected at IMCA-CAT, APS ID17, at a wavelength of 1.000 Å with the A4 210 x 210 mm detector, at a distance of 95 mm with 0.3º oscillation angle for 1200 frames and 3 s exposure, then at 150 mm with 0.5º oscillation for 720 images and 1 s exposures, and finally at 200 mm, 1.0º oscillation for 360 images and 1 s exposure. Images were rebatched to 1º oscillation.

The coordinates for the wild-type ADH–NAD^+–4-iodopyrazole complex (1N92.pdb) were initially used as a molecular replacement model for the H51Q ADH–NAD^+–pyrazole complex because the space groups were the same and the data were of the same resolution.[3] Subsequently, the coordinates for the higher resolution structure of the V292T ADH–NAD^+–pyrazole complex (1N8K.pdb) were used as a model for the H51Q ADH–NAD^+–caprate and the H51Q ADH–NAD(H)–trifluoroethanol complexes.[3] The structures were refined by cycles of restrained refinement with REFMAC 5.8.0267.[4] The REFMAC dictionary for NAD (NAJ) was modified by removing the protonation on the adenine ring and relaxing the restraints on the nicotinamide ring so that planarity or puckering for NAD^+ or NADH could be fitted, such as for the complexes with NAD and pyrazole

or NADH with *N*-cyclohexylformamide or water.[3, 5, 6] The criteria for distinguishing NAD^+ and NADH are described in detail elsewhere.[6] The final refinements with NADH used a dictionary (NAI) with relaxed restraints on the nicotinamide ring.[6, 7] The inhibitors were omitted from the initial refinements of the models, and the residue at position 51 was changed to Ala. Structural models were produced with the program O.[8] The Ala–51 to Gln substitution was made only after clear density for the Gln side chain appeared in the $|F|_o - |F_c|$ map. Many alternative positions and water molecules were identified. The structural models were analyzed with the MolProbity and Parvati servers.[9, 10] Structures of wild-type enzyme complexed with NAD^+ and pentafluorobenzyl alcohol, trifluoroethanol, or 4-iodopyrazole; with NADH and water or *N*-cyclohexylformamide have been determined.[3, 5, 6, 11, 12]

Table S1. Apparent Kinetic Constants for Transient Formation of the H51Q ADH–NAD^+–Pyrazole Complex and Proton Release at Varied pH[a]

	NAD-pyrazole complex, ΔA_{293} [b]			Proton release [c]		
pH	k_{app} (s^{-1})	k_{app}/K_{NAD} ($mM^{-1}s^{-1}$)	K_{NAD} (mM)	k_{app} (s^{-1})	k_{app}/K_{NAD} ($mM^{-1}s^{-1}$)	K_{NAD} (mM)
6.5	1.78 ± 0.04	3.3 ± 0.1	0.53 ± 0.03	1.9 ± 0.2	5 ± 1	0.37 ± 0.14
6.9	1.7 ± 0.6	4.1 ± 0.2	0.43 ± 0.03	2.4 ± 0.3	3.4 ± 0.5	0.73 ± 0.19
7.5	2.42 ± 0.04	7.3 ± 0.2	0.33 ± 0.02	2.8 ± 0.1	8.0 ± 0.5	0.35 ± 0.03
7.9	3.14 ± 0.09	11.9 ± 0.6	0.26 ± 0.02	3.28 ± 0.06	12.6 ± 0.4	0.26 ± 0.01
8.4	4.76 ± 0.09	26 ± 1	0.18 ± 0.01	4.55 ± 0.09	19.8 ± 0.8	0.23 ± 0.01
8.6	8.7 ± 0.2	36 ± 1	0.24 ± 0.01	9.3 ± 0.3	32 ± 1	0.29 ± 0.02
8.7	9.7 ± 0.1	44 ± 1	0.22 ± 0.01	7.9 ± 0.4	39 ± 4	0.20 ± 0.03
9.0	18 ± 1	68 ± 6	0.26 ± 0.04	18 ± 1	54 ± 4	0.33 ± 0.04
9.4	38 ± 1	103 ± 5	0.36 ± 0.03	60 ± 10	120 ± 30	0.55 ± 0.24
9.8	46 ± 2	119 ± 7	0.39 ± 0.04	90 ± 10	160 ± 25	0.54 ± 0.15

[a] For reaction at each pH, the apparent kinetic constants were determined from the fit of the observed rate constants at varied concentrations of NAD^+ and fixed (10 mM) concentration of pyrazole (e.g., data in Figure 1 in the main text) with the equation $k_{obs} = k_{app}[NAD^+]/(K_{NAD} + [NAD^+])$[13], where k_{app} is the apparent maximum rate constant and K_{NAD} corresponds to the binding constant. [b] Formation of the enzyme–NAD^+–pyrazole complex was monitored by an increase in absorbance at 293 nm. [c] Proton release was monitored by a decrease in absorbance of chlorophenol red (pH 6.5, A_{575}), phenol red (pH 6.9–8.4, 8.7: A_{559} nm) and thymol blue (pH 8.6, 9.0 and 9.4: A_{596} nm) and by an increase in absorbance of thymolphthalein (pH 9.8, A_{595} nm).

Table S2. pH Dependences for Reactions of H51Q ADH with NAD^+ and Pyrazole[a]

Reaction, parameter (units)	pK values E (E complex)	Limiting values low pH and high pH
$k_{app, \text{proton release}}$ (s^{-1})	10.1 ± 0.2	k_a 1.7 ± 0.3
	(7.8 ± 0.2)	k_b 300 ± 100
$k_{app, NAD^+ \text{binding}}$ (s^{-1})	9.6 ± 0.1	k_a 1.7 ± 0.3
	(7.9 ± 0.1)	k_b 80 ± 20
$k_{app}/K_{NAD, \text{proton release}}$ ($mM^{-1}s^{-1}$)	9.3 ± 0.1	k_a 4 ± 2
	(7.6 ± 0.1)	k_b 220 ± 20
$k_{app}/K_{NAD, NAD^+ \text{binding}}$ ($mM^{-1}s^{-1}$)	9.0 ± 0.1	k_a 3.0 ± 0.2
	(7.5 ± 0.1)	k_b 140 ± 20
$K_{i, \text{pyrazole}}$ (μM)[b]	9.0 ± 0.1	$K_{d,HE}$ 150 ± 40
	(7.8 ± 0.2)	$K_{d,E}$ 10 ± 1

[a] The data in Supporting Information Table S1 were fitted to the equation for a simple pH dependence where two forms of enzyme, E and EH (protonated), are related by the pK value, and k_a represents the rate constant for reaction of HE, and k_b represents the rate constant for reaction of E. If both forms react, the "WAVL" equation applies: $k_{obs} = (k_a + k_b K/[H^+])/(1 + K/[H^+])$. The principle of linked equilibria ($k_a K_1 = k_b K_2$, where k_a is the rate constant for the protonated free enzyme, k_b is the rate constant for the unprotonated enzyme, K_1 is the dissociation constant for deprotonation of the free enzyme, and K_2 is the dissociation constant for the enzyme complexes provides an implied pK for the enzyme complexes (the low pK). The data are listed in Supporting Information, Table S1 (k_{app} and k_{app}/K_{NAD^+}) and Table S3 (K_i). [b] The buffers contained 10 mM sodium salts of MOPS, TAPS, and AMPSO with 33 mM Na_2SO_4. Similar results were obtained in the pyrophosphate/phosphate buffer system. The association constant (reciprocal of the K_i value) was fitted to obtain the pK value for the enzyme-NAD^+ complex.

Table S3. pH Dependence for Steady-state Competitive Inhibition by Pyrazole of Ethanol Oxidation by H51Q ADH[a]

pH	V_1/E_t (s^{-1})	K_b, ethanol (mM)	K_i, pyrazole (μM)
6.7	0.34 ± 0.03	1.9 ± 0.3	160 ± 50
7.2	0.70 ± 0.04	1.4 ± 0.2	120 ± 20
8.0	1.5 ± 0.1	0.71 ± 0.03	63 ± 5
8.2	1.8 ± 0.4	0.60 ± 0.04	37 ± 4
8.6	2.2 ± 0.2	0.32 ± 0.02	28 ± 2
9.0	2.8 ± 0.3	0.30 ± 0.01	19 ± 2
9.3	2.7 ± 0.4	0.26 ± 0.01	11 ± 1
9.5	2.8 ± 0.4	0.30 ± 0.01	12 ± 1
9.7	3.6 ± 0.4	0.29 ± 0.01	11 ± 1

[a] The buffers contained 33 mM Na_2SO_4, with sodium salts of 10 mM MOPS, TAPS, and AMPSO, at 25 °C, with 2 mM NAD^+, and four to five concentrations of ethanol varied over a 5–15-fold range (0.05–6.4 mM) and five concentrations of pyrazole over an 8-fold range (2–200 μM). The concentrations were adjusted as the pH was varied. Duplicate or triplicate assays of NADH production were determined at 340 nm. The data were fitted with the equation for competitive inhibition.[13] The turnover numbers (V_1/E_t) and Michaelis constants (K_b) approximate the values obtained in previous studies and with the pH dependence determined in the pyrophosphate buffer system. The pH dependence for inhibition fits a WAVL function, with pK values and finite, limiting

values at low and high pH (Supporting Information Table S2). Pyrazole is a competitive inhibitor for oxidation of varied concentrations of ethanol by wild-type ADH with a K_i of 0.2 μM, and is uncompetitive against varied concentrations of NAD^+, consistent with binding of pyrazole to the enzyme-NAD^+ complex.[14] The initial velocity progress curves show a transition to a slower rate during the first few minutes, presumably due to tighter binding as the initial enzyme–NAD^+–pyrazole deprotonates, and the earliest phase provides the pH dependence for K_i value for the initial complex. The pH dependences for the reaction of ethanol were fitted with pK values of 8.6 ± 0.1 and k_b = 12 ± 1 $mM^{-1}s^{-1}$ for V_1/E_tK_b and 7.6 ± 0.1 and k_b = 2.6 ± 0.3 s^{-1} for V_1/E_t. Similar results were obtained in pyrophosphate/phosphate buffers.

Stoichiometry of Proton Release. The number of protons released per enzyme subunit ($[H^+]/[E]$) during reaction of the H51Q enzyme with NAD^+ and pyrazole was determined from the transient kinetics and direct potentiometric titrations (Supporting Information Figure S1A). The proton stoichiometry exhibits a bell-shaped pH dependence, and the data were fitted with the equation for the mechanism in main text Scheme 3, where both the free enzyme and the enzyme–NAD^+–pyrazole complex ionize in the observable pH range.

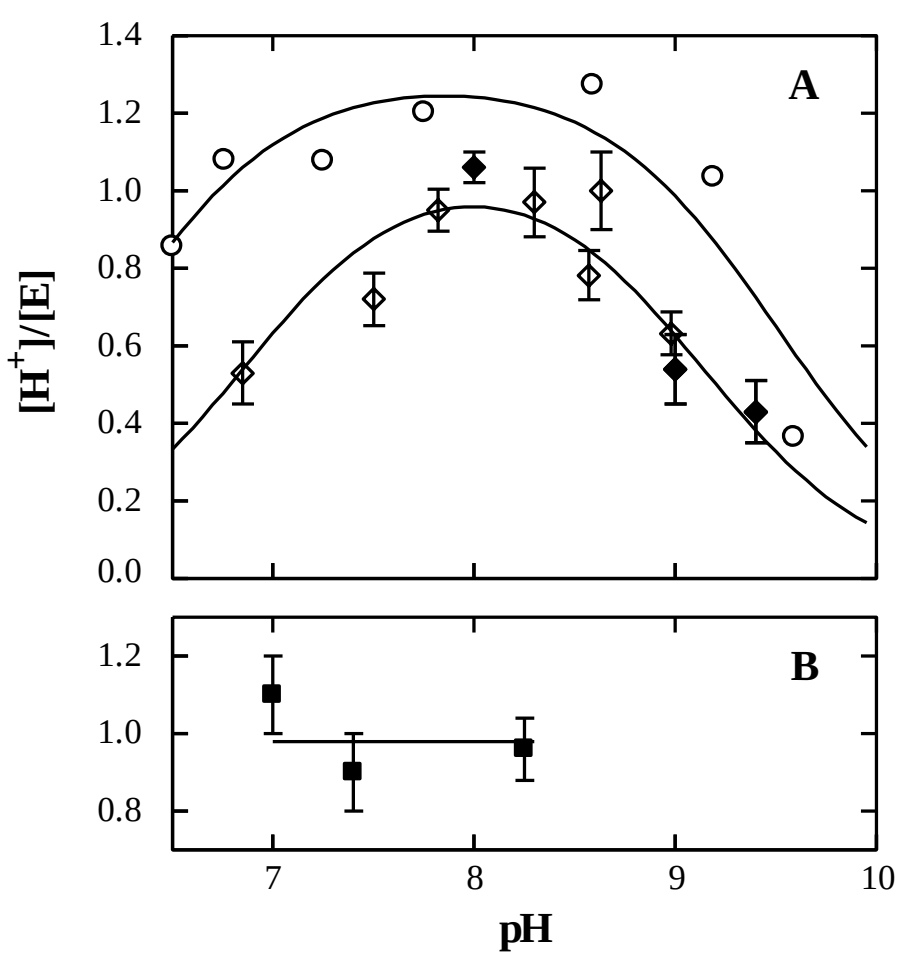

Figure S1. The pH dependence of proton stoichiometry during formation of (A) the enzyme–NAD^+–pyrazole and (B) the enzyme–NAD^+–trifluoroethanol complexes with H51Q ADH. (A) The number of protons released was determined from the amplitudes of transient progress curves for reaction of the enzyme (10–20 μN) with NAD^+ and either fixed (◇, Supporting Information Table S1) or varied (◆, main text Table 2) concentrations of pyrazole in the presence of different pH indicators, and by potentiometric titration of the enzyme (106 μN) with NAD^+ (0–0.45 mM) in the presence of 10 mM pyrazole (○). The data were fitted with the equation $[H^+]/[E]_{obs} = ([H^+]/[E]_{max})/(1/(1 + K_E/[H^+]) - 1/(1 + K_{E\text{-}NAD\text{-}Pyz}/[H^+]))$, where pK_E is estimated to be 9.2 ± 0.2, and $pK_{E\text{-}NAD\text{-}Pyz}$ is 6.5 ± 0.2 for each curve, and $[H^+]/[E]_{max}$ is 1.3 ± 0.3 or 1.1 ± 0.1. (B) The number of protons released was determined from the amplitudes of transient progress curves for reaction of 10–20 μN enzyme with NAD^+ and varied concentrations of trifluoroethanol (■, main text Table 3). The line represents the average value of 0.98. Reactions were carried out in 33 mM Na_2SO_4, 0.25 mM EDTA, at 25 °C under N_2 atmosphere.

The pH dependence suggests that the pK of ~9.2 of some group on the free enzyme (such as the zinc-water) is shifted to a pK of ~ 6.5 when the NAD–pyrazole adduct forms. The pH dependence for proton release from wild-type ADH was determined from transient proton release and potentiometric

studies over the pH range of 6.5–9.5 and showed only a pK of ~9.5[15], whereas a previous potentiometric study over the pH range of 6–10 showed pK values of 9.5 and 6.0.[16] The p*K* value for the pyrazole proton in the ternary complex with the wild-type enzyme is estimated to be less than 4 or 5[15, 17], and therefore the ternary complex should only have the deprotonated form of pyrazole in the pH range of 6.5–10.[16] The rate constant for dissociation of pyrazole increases with decreasing pH, indicating that protonation disrupts the binding.[17] The pK value for pyrazole in the complex with NAD^+ could be elevated to a value above 6 with the H51Q enzyme. However, the structure of the NAD^+–pyrazole complex with the H51Q enzyme is the same as that with wild-type enzyme (this study, 8G2X.pdb), and the extinction coefficient for the ternary complex (at 293 nm) with H51Q ADH was independent of pH range in the range of 6.2–9.8 at 25 ºC. One proton is released per enzyme subunit during the transient reaction of the H51Q enzyme with NAD^+ and trifluoroethanol at pH values from 7.0 to 8.25 (Figure S1B).

Table S4. Microscopic Rate Constants for Reactions of H51Q ADH with Varied Concentrations of NAD$^+$ and Pyrazole for Either Isomerization of the Enzyme–NAD$^+$ Complex or the Enzyme–NAD$^+$–Pyrazole Complex

1. Proton release associated with E-NAD$^+$ isomerization (step 2)[a]

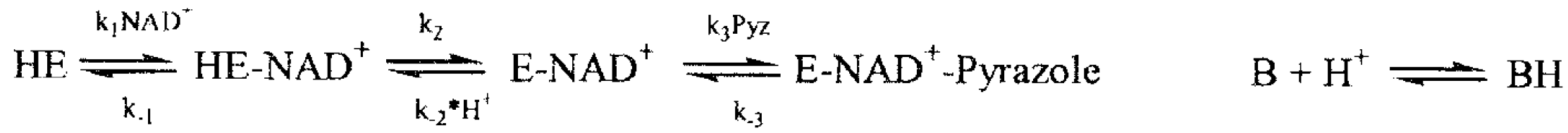

pH	k_1 ($\mu M^{-1}s^{-1}$)	k_{-1} (s^{-1})	k_2 (s^{-1})	k_{-2} ($nM^{-1}s^{-1}$)	pK_2 $-\log(k_2/k_{-2}$	k_3 ($\mu M^{-1}s^{-1}$)
8.0[b]	0.085 ± 0.003	10.1 ± 0.4	4.00 ± 0.02	180±120	10.6	0.44±0.30
8.0[c]	0.051 ± 0.001	3.5 ± 0.1	3.64 ± 0.03	390±200	11.0	0.70±0.40
8.0[d]	0.062 ± 0.001	5.3 ± 0.2	3.90 ± 0.02	500±300	11.1	1.1±0.6

[a] These simulations (Kinsim/Fitsim) used sixteen time courses with varied concentrations of both pyrazole (2, 5, 9.8 and 20 mM) and NAD$^+$ (0.047–0.8 mM) at pH 8.0. The data are the same as used for Table 2 in the main text, and in part 2 below. The rate constant for dissociation of the E–NAD$^+$–pyrazole complex, k_{-3}, was fixed at 0.02 s^{-1}. The rate constants for the protonation of the buffer (B) were fixed to match the pK, $k_4 = 1 \times 10^{10}$ $M^{-1}s^{-1}$ and $k_{-4} = 126$ s^{-1} for phenol red. The total concentration of phenol red was 10 μM, but buffering by protein was accounted for by simulation with 43.6 μM [B] and 36.4 μM [BH], as estimated by titration of the experimental solution with HCl. The simulations fit the experimental data very well, $R^2 \geq 0.999$. [b]Monitored at A_{293} for formation of the enzyme–NAD–pyrazole complex. Rate constants for step 1 (k_1/k_{-1}) are highly correlated, as are constants k_{-2} and k_3. [c] Monitored for proton release at A_{559}. [d]Monitored at both A_{293} and A_{559}. Rate constants k_1 and k_{-1} are highly correlated, as are k_{-2} and k_3. The dissociation constant for NAD$^+$ ($K_{ia} = k_{-1}k_{-2}[H^+]/k_1(k_2 + k_{-2}[H^+])$) is estimated by the ratio k_1/k_{-1}, to be 90 μM.

2. Proton release associated with deprotonation of E-NAD$^+$-pyrazole (step 3)[a]

k_1 k_2 k_3 k_4

E === E-NAD$^+$ === E-NAD$^+$-PYZ === E-NAD-PYZ + H$^+$ H + B == BH

k_{-1} k_{-2} k_{-3} [H$^+$] k_{-4}

pH	k_1 ($\mu M^{-1}s^{-1}$)	k_{-1} (s^{-1})	k_2 ($\mu M^{-1}s^{-1}$)	k_{-2} (s^{-1})	k_3 (s^{-1})	k_{-3} ($\mu M^{-1}s^{-1}$)
8.0 [b]	0.020±0.001	1.68±0.04	0.3±0.2	4500±2800	8.4±0.2	0.80 ± 0.04
8.0 [c]	0.029±0.001	2.33±0.06	0.6±0.4	3900±2700	3.63±0.04	1.91 ± 0.04
8.0 [d]	0.024±0.002	1.90±0.04	2.8±1.4	27000±14000	5.48±0.08	0.91 ± 0.04

[a] The data were the same as used above. [b] Monitored at 293 nm. Rate constants for step 2 are highly correlated. [c] Monitored at 559 nm. [d] Monitored by absorbance at 293 and 559 nm for proton release. K_d ($= k_{-1}/k_1$) for NAD$^+$ is ~80 μM; pK_3 is 5.5; and $K_{iz} = k_{-2}k_{-3}[H^+]/k_{+2}(k_{-3}[H^+] + k_{+3})$ for pyrazole is 15 μM, which is similar to the steady-state results (Table S3).

Table S5. Microscopic Rate Constants for Binding of NAD^+ and Trifluoroethanol to H51Q ADH for Either Isomerization of the Enzyme–NAD^+ Complex or the Enzyme–NAD^+–Trifluoroethanol Complex

Simulations of multiple transients of protein fluorescence (X = amplitude) or proton release.[a]

1. Simplest bimolecular association.

$$E \rightleftharpoons E\text{–}NAD^+ \rightleftharpoons E\text{–}NAD^+\text{–TFE}$$

pH	k_1 $mM^{-1}s^{-1}$	k_{-1} s^{-1}	k_2 mM^{-1} s^{-1}	k_{-2} s^{-1}	X_E mM^{-1}	X_{EA} mM^{-1}	X_{FAT} mM^{-1}	Mean SQ
7.4	1020 ± 10	14 ± 1	7.8 ± 0.1	3.9 ± 0.2	283 ± 1	226 ± 1	183 ± 1	5.0 E–5

2a. Isomerization of E-NAD^+ before binding of trifluoroethanol

$$E \rightleftharpoons E\text{-}NAD^+ \rightleftharpoons {}^*E\text{-}NAD^+ \rightleftharpoons {}^*E\text{-}NAD^+\text{-TFE}$$

pH	k_1 $mM^{-1}s^{-1}$	k_{-1} s^{-1}	k_2 s^{-1}	k_{-2} s^{-1}	k_3 $mM^{-1}s^{-1}$	k_{-3} s^{-1}	X_E	X_{EA}	X_{FA}	X_{FAT}	Mean SQ
7.0	1340 ± 50[a]	74 ± 12[a]	280 ± 10	300 ± 100[b]	26 ± 5[b]	0.6 ± 0.4	303	245	212	194	5.3 E–5
7.4	1760 ± 80[a]	120 ± 20[a]	270 ± 10	200 ± 40[b]	22 ± 2[b]	3 E–7	283	238	188	178	4.7 E–5
8.2	2030 ± 230[a]	280 ± 60[a]	370 ± 20	220 ± 50[b]	19 ± 2[b]	3 E–6	266	245	174	172	3.0 E–5

2b. Proton release at step 2.

pH	k_1 $\mu M^{-1}s^{-1}$	k_{-1} s^{-1}	k_2 s^{-1}	k_{-2} $nM^{-1}s^{-1}$	k_3 $mM^{-1}s^{-1}$	k_{-3} s^{-1}	pK_2	$[H^+]$ k_{-2} s^{-1}	Mean SQ
7.4	76 ± 24[a]	8.0 ± 2.4 E3[a]	231 ± 2	15 ± 0.5[b]	65 ± 2[b]	5.4 ± 0.1	7.8	600	2.6 E–7

3a. Isomerization of ternary complex with NAD^+ and trifluoroethanol.

$$E \rightleftharpoons E\text{–}NAD^+ \rightleftharpoons E\text{–}NAD^+\text{–TFE} \rightleftharpoons {}^*E\text{–}NAD^+\text{–TFE}$$

pH	k_1 $mM^{-1}s^{-1}$	k_{-1} s^{-1}	k_2 $mM^{-1}s^{-1}$	k_{-2} s^{-1}	k_3 s^{-1}	k_{-3} s^{-1}	X_E	X_{EA}	X_{EAT}	X_{FAT}	Mean SQ
7.0	1140 ± 20	57 ± 5	60 ± 40[a]	560 ± 450[a]	176 ± 14	3.2 ± 0.7	303	234	233	193	5.1 E–5
7.4	1300 ± 20	65 ± 5	680 ± 1400[a]	8100 ± 17000[a]	200 ± 10	1.9 ± 0.4	284	218	231	180	4.7 E–5

3b. Proton release at step 3.

pH	k_1 $mM^{-1}s^{-1}$	k_{-1} s^{-1}	k_2 $mM^{-1}s^{-1}$	k_{-2} s^{-1}	k_3 s^{-1}	k_{-3} μM^{-1} s^{-1}	pK_3	$[H^+]$ k_{-3} s^{-1}	Mean SQ
7.4	1680 ± 20[a]	142 ± 4[a]	31.5 ± 0.8[b]	190 ± 10[b]	161 ± 2	105 ± 2	5.8	4.2	2.0 E–7

[a] The data are the same as those used for Table 3 in the main text with varied concentrations of NAD^+ and trifluoroethanol. "*E" or "F" represent the conformation after isomerization and deprotonation. High covariance is indicated for the pairs indicated by [a] or [b]. "T" represents TFE, 2,2,2-trifluoroethanol. For the fluorescence data, the rate constants and output factors (X) were fitted together, and errors on the estimates of X were usually < ± 2. See next section for the history linking the quenching of fluorescence to the binding of NAD^+ and the conformational change. For mechanism 2, the dissociation constant for NAD^+ (K_{ia}) is calculated by $k_{-1}k_{-2}/k_1(k_2 + k_{-2})$, and $k_{on,NAD+} = k_1k_2/(k_{-1} + k_2)$. The dissociation constant for trifluoroethanol (K_D) is corrected for the isomerization of the enzyme–NAD^+ complex with $k_{-3}(k_2 + k_{-2})/k_2k_3$. For mechanism 3, the dissociation constant for trifluoroethanol is calculated from $k_{-2}k_{-3}/k_2(k_3 + k_{-3})$. Protonation steps are bimolecular, and the calculated first order rate constant is somewhat larger than the value estimated from the fluorescence data.

Linking the binding of NAD^+ to quenching of protein fluorescence and the conformational change. Simulations of the binding of NAD^+ and trifluoroethanol monitored by protein fluorescence show that the apparent magnitudes of quenching change progressively during the overall reaction and provide evidence for the conformational change of the enzyme-NAD^+ complex (Table 4 in Ref.[15] and Supporting Information Table S5). Binding of adenosine diphosphoribose or nicotinamide ribose does not quench the fluorescence, and the complex with ADPR has the open conformation, indicating that the whole coenzyme (NAD^+ or NADH) is required for the conformational change.[6, 18] The pH dependence of the quenching is complicated.[6, 19-23] Trp-314 is involved.[24-26] Fluorescence of the apoenzyme is quenched by raising the pH, with an apparent pK of 9.6–9.8.[21, 27] Binding of NAD^+ also quenches the fluorescence, and the pH dependence of the change corresponds to the pK value of ~9.6 for the group of the apoenzyme that controls the binding and is shifted to a pK of ~7.6 in the enzyme-NAD^+ complex.[21] Assigning the responsible group on the protein based on the pH dependence has been controversial.[16, 23, 27] One study suggested that the pH dependence of the fluorescence quenching was associated with the ionization of Tyr-286 with a pK of 9.8, which shifts to 7.6 in the enzyme-NAD^+ complex, but the X-ray structures do not show much change in the environments of Tyr-286 or Trp-314 upon formation of complexes with coenzymes.[6, 21, 26, 28] Other possible candidates for the group with the pK of ~9.8 that quenches fluorescence of the apoenzyme -- zinc-water, Cys-46, or Lys-228 -- are apparently not responsible.[23, 27] His-51 was also a candidate.[16] A robust reconciliation of the different studies is that there are two dynamic quenching mechanisms of Trp-314, one involving the pH dependent binding of NAD^+ and the other the ionization of Tyr-286.[27] At pH 8, quenching is about 3% by ionization of Tyr-286, 31% by formation of the NAD^+–pyrazole complex, and 54% with the NAD^+–trifluoroethanol complex. Thus, most of quenching at pH 8 is associated with the formation of complexes and effects on Trp-314, rather than ionization of Tyr-286. However, when Trp-314 is replaced by Leu-314 in a triply-substituted ADH, protein fluorescence of the apoenzyme is not quenched by raising the pH to 10, but formation of complexes with NAD^+ and pyrazole or trifluoroethanol quenches by 15–20%, apparently due to Trp-15, which is on the surface of the protein.[26]

Table S6. Kinetic Constants for Oxidation of Ethanol Determined by Transient and Steady State Kinetics in 10 mM Sodium TAPS, 33 mM Na_2SO_4, pH 8 Buffer [a]

	Wild-type ADH			H51Q ADH
Kinetic Constant	Burst[b]	Steady-state[b]	Steady-State[c]	Steady-State[d]
V_1/E_t (s^{-1})	150 ± 6	3.9 ± 0.1	3.3 ± 0.2	1.7 ± 0.1
K_a, NAD^+ (mM)	0.18 ± 0.03	0.031 ± 0.009	0.021 ± 0.005	0.098 ± 0.009
K_b, ethanol (mM)	3.9 ± 0.3	0.26 ± 0.05	0.21 ± 0.04	0.64 ± 0.06
K_{ia}, NAD^+ (mM)	0.17 ± 0.04	1.0 ± 0.3	0.23 ± 0.05	0.11 ± 0.01

[a] For comparison to the proton release data, which used weakly buffered 33 mM Na_2SO_4, and other experiments that used sodium phosphate buffers, these data (duplicate assays) were collected using 10 mM sodium TAPS buffer, pH 8.0, with 33 mM Na_2SO_4 and 0.25 mM EDTA, at 25 ºC, by varying the concentrations of both NAD^+ (A) and ethanol (B), with fitting with the Sequential Bi mechanism, $v = V_1AB/(K_{ia}K_b + K_aB + K_bA + AB)$.[13] [b] Transient kinetics used the BioLogic SFM3 stopped-flow with which a distinct exponential *Burst* phase was followed by a *Steady-state* slope phase. The enzyme concentration was 9.8 μN; NAD^+ concentrations were varied at 0.07, 0.23, 0.87, and 2.9 mM, and nine ethanol concentrations from 0.45–10 mM were used. [c] The steady-state data used five concentrations of NAD^+ from 20–310 μM and five concentrations of ethanol from 0.14–3.0 mM with 2 μN enzyme with a Cary 118C spectrophotometer. [d] Reactions of six concentrations of NAD^+ from 49–470 μM and six concentrations of ethanol from 0.24–2.5 mM were studied with the

spectrophotometer. With the stopped-flow instrument, the burst phases of the progress curves with H51Q ADH were not well-defined, with an estimated rate constant of ~8 s^{-1} at 2.5 mM NAD^+ and varied concentrations of ethanol (K_b 0.8 mM), and a steady-state turnover number of ~1 s^{-1}.

Table S7. Simulations of the Catalytic Reaction with Isomerization of E-NAD⁺ (EA = FA, Step 2), or E-NAD⁺-Alcohol (FAB = FAX, Step 4), and Hydride Transfer (Step 5)[a]

1 2 3 4 5 6 7 8 9

E = EA = FA = FAB = FAX = FPQ = FQ = E B + H^+ = BH^+ HOH = H^+ + OH^-

Enzyme	WT FA[b]	H51Q FA[c]	H51Q FA[d]	H51Q FAX[e]	H51Q FAX[f]	H51Q H^+/H^- [g]
k_1 ($\mu M^{-1}s^{-1}$)	*45*	*4.9* ± 0.6	*2.8*	*2.8*	*1.8*	*1.8*
k_{-1} (s^{-1})	*2300*	*1000* ± 100	*200*	*200*	*300*	*300*
k_2 (s^{-1})	*620*	*19* ± 3	**11.0 ± 0.1**	NR	*280*	*280*
k_{-2} (s^{-1})	*64*	*2100* ± 300	**2200±600**	NR	*250*	*250*
k_3 ($\mu M^{-1}s^{-1}$)	0.32	1.2 ± 0.02	18 ± 4	0.27 ± 0.09	0.82 ± 0.07	0.59 ± 0.06
k_{-3} (s^{-1})	4900	56 ± 1	154 ± 5	480 ± 160	780 ± 70	380 ± 40
k_4 (s^{-1})	8000	NR	NR	**810 ± 550**	**5800 ± 4200**	**concerted**
k_{-4} (s^{-1})	1300	NR	NR	**970 ± 660**	**6700 ± 4900**	**H+/H–**
k_5 (s^{-1})	520	50 ± 10	71 ± 2	13.1 ± 0.5	14.6 ± 0.3	**71 ± 4**
k_{-5} (s^{-1})	530	650 ± 60	111 ± 2	73 ± 2	75.4 ± 0.6	**167 ± 6**
k_6 (s^{-1})	61	340 ± 80	16.7 ± 0.4	570 ± 140	300 ± 20	18.1 ± 0.7
k_{-6} ($\mu M^{-1}s^{-1}$)	0.22	0.12 ± 0.01	0.0752±0.0003	0.5 ± 0.1	0.33 ± 0.02	0.071 ± 0.001
k_7 (s^{-1})	*5.5*	*4.6* ± 0.2	*4.0*	*4.0*	*4.0*	*4.0*
k_{-7} ($\mu M^{-1}s^{-1}$)	*11*	*8.0* ± 1	*6.7*	*6.7*	*6.7*	*6.7*
pK			pK_2 10.3	pK_4 8.1	pK_4 8.1	pK 8.4
Fratio			1.74 E+7	4.82E+6	1.77E+7	5.94E+6
MeanSQ			5.10 E–6	1.84E–6	5.02E-6	2.00E–6

[a] Symbols: E open conformation, F postulated closed conformation; A, NAD^+; B, ethanol; X, alkoxide; P, acetaldehyde; Q, NADH. FA and FAX represent the deprotonated complexes for one mechanism or the other. Units are $\mu M^{-1}s^{-1}$ for bimolecular steps and s^{-1} for unimolecular steps. "NR" means "not relevant" for this mechanism. Rate constants in italics for coenzyme binding were determined in separate experiments and fixed for these simulations. The dissociation constant for NAD^+ calculated from the rate constants is ~ 70 μM. Proton release data were used for the four columns on the right side, as indicated in bold face, where the bimolecular rate constant for the reverse protonation (k_{-2} or k_{-4}) has been multiplied by the concentration of H^+ at pH 8. The present study used eight transients (2.5 mM NAD^+ and 0.95, 1.3, 2.2 and 6.3 mM ethanol) for oxidation and proton release and four transients for acetaldehyde reduction (0.1 mM NADH and 0.4, 0.5, 0.9, and 2.1 mM acetaldehyde, truncated to the first 0.15 s so that multiple turnovers did not change the pH too much) in 33 mM sodium sulfate, pH 8, at 25 ºC. The concentration of the indicator, phenol red, was 10 μM, but buffering by the protein led to effective concentrations of 55.8 μM basic form and 46.6 μM acidic form as determined by titration of the reaction solution. Rate constants were fixed at $k_8 = 1 \times 10^{10}$ $M^{-1}s^{-1}$ and $k_{-8} = 126$ s^{-1} for the indicator pK of 7.9. The ionization of water was also included as a reservoir for protons, with rate constants fixed at $k_9 = 2.5 \times 10^{-5}$ s^{-1} and $k_{-9} = 1.4 \times 10^{11}$ $M^{-1}s^{-1}$. [b] Wild-type enzyme, forward and reverse hydride transfer reactions, estimated errors <10% except up to 50% for steps 3 and 4, which are speculative.[29] [c] H51Q ADH, four forward and four reverse hydride transfer reactions in 33 mM sodium phosphate buffer, pH 8.[1] [d] Proton release coupled to isomerization of enzyme-NAD^+ complex. The simulated transients fit the data well ($R^2 = 0.9999$), but k_{-2} and k_3 are highly correlated (0.99). [e] The same number of rate constants were fitted for these data in order to compare with the fits for H51Q FA. Rate constants for steps 3, 4, and 6 are highly correlated. Rate constants k_{-4} and k_{-5} are larger than 34 s^{-1} in 33 mM sodium sulfate determined with

0.1 mM NADH and varied concentrations of acetaldehyde (K_m 0.49 ± 0.06 mM) in the present study. (The turnover number for acetaldehyde reduction is 54 s^{-1} in 33 mM sodium phosphate buffer.[30]) [f]Rate constants for binding of NAD^+ were estimated from the simulations with trifluoroethanol (Table 5S.2) and fixed. Calculated overall rate constants are k_{on} = 0.87 $uM^{-1}s^{-1}$, and k_{off} = 68 s^{-1}. Rate constants for steps 3, 4 and 6 are highly correlated, and various values that fit the ratios (k_3/k_{-3} and k_4/k_{-4}) simulate the reactions well, with K_3 = 0.9 mM and K_6 = 0.9 mM. The simulations fit the data well (R^2 = 1.000). [g] Concerted H^+/H^- transfer, with steps 4 and 5 combined. R^2 = 0.9998, but k_3/k_{-3} are correlated (0.97), and k_{-5} is negatively correlated (–0.97) with k_3 and k_{-3}.

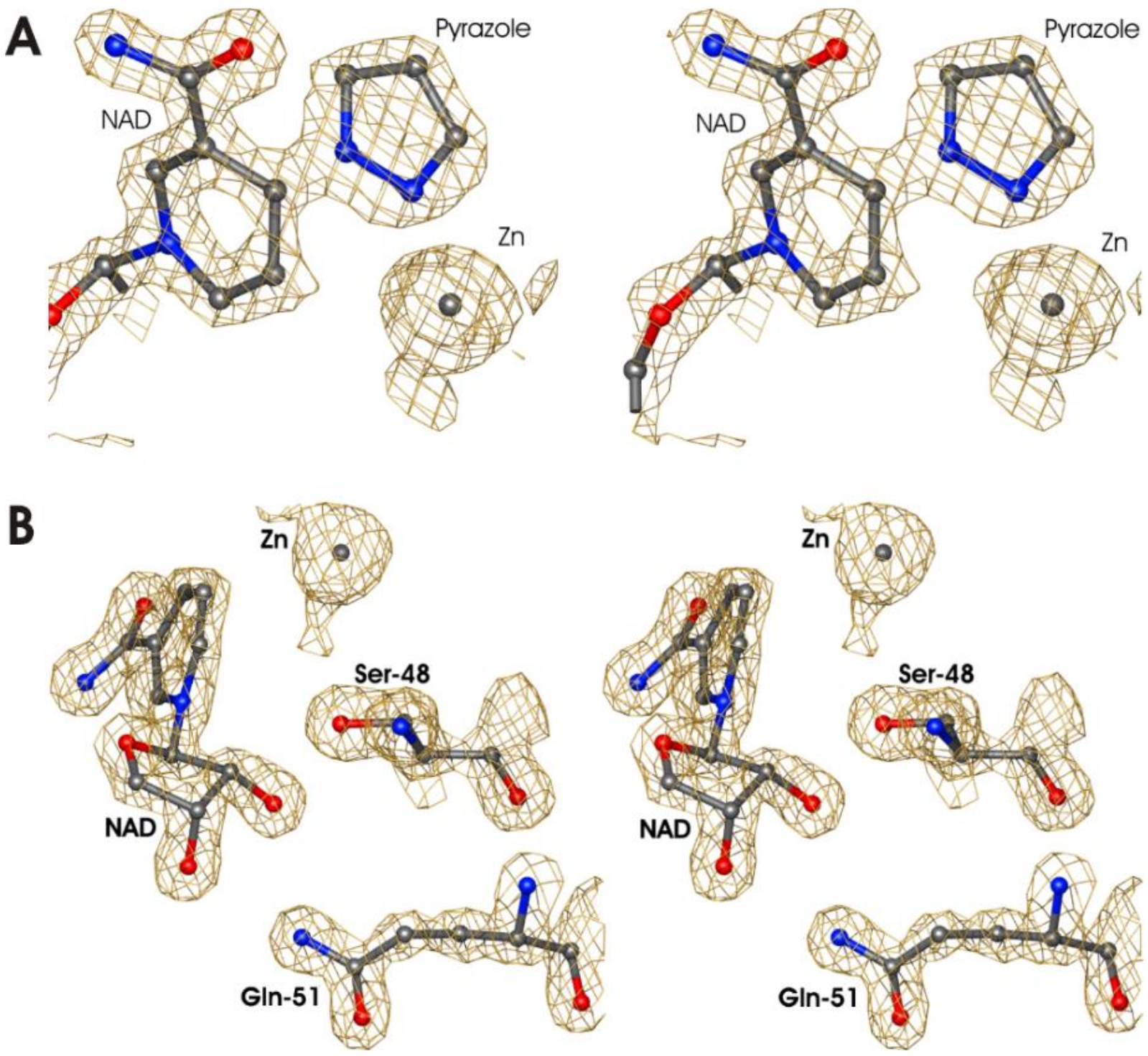

Figure S2. Electron density map defining the positions of pyrazole and Gln-51 in the H51Q ADH–NAD^+–pyrazole complex (8G2X.pdb). (A) Position of the bound pyrazole. (B) Position of Gln-51.

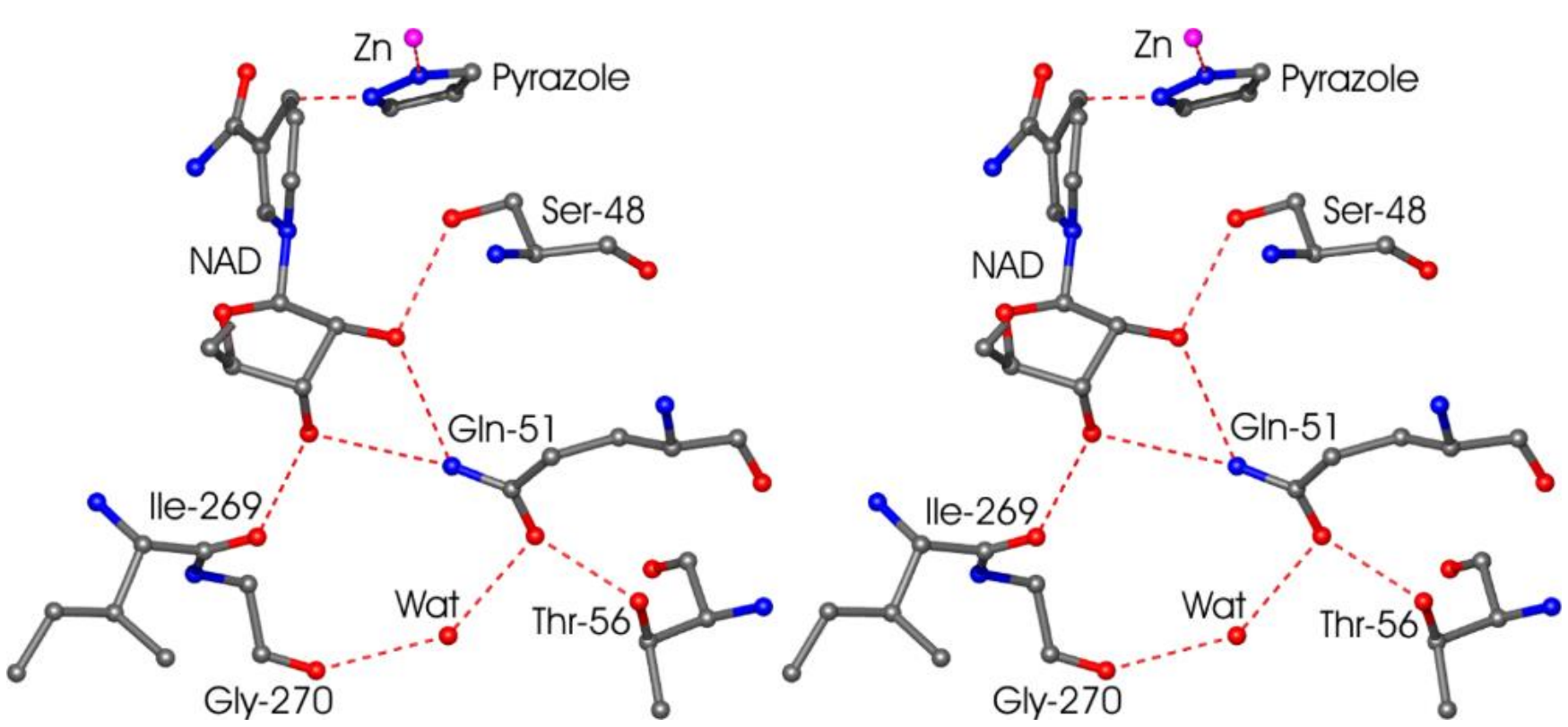

Figure S3. Hydrogen-bonding interactions in the active site of the H51Q ADH–NAD^+–pyrazole complex. Dashed lines represent hydrogen bonds that were inferred from the close contacts (2.6–3.2 Å), and the bond lengths are summarized in Tables S8 and S9.

Table S8. Interactions for Hydride and Proton Transfer in the Active Sites of Complexes of ADHs[a]

Enzyme, PDB entry	Ligand to Zn	Zn–O	O–Ser48 OG	Ser48 OG–NAD O2D	O2D–NE2	C–C4N NAD
WT, 4DWV	PFB	1.97	2.50	2.65	3.07	3.36
WT, 4DXH	TFE	1.97	2.48	2.66	3.07	3.38
H51Q, 8G4V	PFB	1.92	2.59	2.68	3.18	3.22
H51Q, 8G2L	TFE	2.04	2.45	2.65	3.10	3.38
H51Q, 8G2S	caprate	2.06	2.70	2.66	3.16	3.50
H51Q, 8G39	CXF	2.13	2.75	2.74	3.17	3.55
H51Q, 8G41	water	2.09	2.67	2.70	3.26	-----
H51Q, 8G2X	pyrazole	2.11 (N1)	3.2 (N1,2)	2.72	3.10	1.62 (N2)

[a] Average of the distances in Å units of two subunits, with averaged values for the alternative conformations of TFE or caprate. Abbreviations: PFB, 2,3,4,5,6-pentafluorobenzuyl alcohol; TFE, 2,2,2-trifluoroethanol; CXF, *N*-cyclohexylformamide. The complexes with CXF and water contain NADH. The estimated errors in the coordinates and the range of values suggest that differences larger than 0.1 Å might be significant. The "O" is for the ligand, which is replaced by N1 in pyrazole. "NE2" is for either Gln-51 or His-51. "C" is the carbon of the ligand that mimics the methylene carbon in a reactive alcohol, but N2 of pyrazole forms a partial covalent bond with C4N of NAD.[3]

Table S9. Interactions of Gln-51 in the Active Sites of Complexes of H51Q ADH[a]

Enzyme, PDB entry "A" and/or "B" conformation	Ligand to catalytic zinc	NAD form	NE2–NAD O2D	NE2–NAD O3D	NE2–Val-294 O	OE1–OH_2	OE1–Thr-56 OG1
H51Q, 8G2X	pyrazole	NAD	3.16	3.20	3.00	2.73	2.58
H51Q, 8G2L	TFE	NAD(H)	3.10	3.13	3.05	2.71	2.66
H51Q, 8G2S	caprate	NAD^+	3.16	3.19	3.04	2.71	2.70
H51Q, 8G39	CXF	NADH	3.17	3.22	3.02	2.72	2.68
H51Q, 8G4V "A"	PFB	NAD^+	3.18	3.25	3.00	2.74	2.75
H51Q, 8G41 "A"	water	NADH	3.26	3.20	3.22	2.72	2.68
Alternative "B" position for Gln-51			OE1–NAD O2D	OE1–NAD O3D	NE2–Val-294 O	NE2–OH_2	NE2–NAD O3D
H51Q, 8G4V "B"	PFB	NAD^+	2.50	3.76	3.38	3.23	3.22
H51Q, 8G41 "B"	water	NADH	2.39	3.76	3.20	3.37	2.68
H51Q/K228R 1QV6[b]	2,4-DFB	NADH	2.56	3.56	3.16	3.19	3.19
H51Q/K228R 1QV7[b]	2,3-DFB	NADH	2.90	3.20	3.47	3.16	3.16

[a] Average of the distances in Å units of two subunits. The estimated errors in the coordinates and the range of values suggest that differences of 0.1 Å are significant. The complexes with PFB and water have both conformations in each active site. The other structures have only one major conformation.
[b] For two subunits after further refinement of structures with difluorobenzyl alcohols (DFB) and NADH at 1.8 Å resolution.[1]

Table S10. Alternative Interactions (Å) of Benzyl Alcohols in ADH–NADH Complexes[a]

Enzyme, PDB	Benzyl alcohol, Coenzyme		Zn–O1	Ser48 OG–O1	Ser48 OG–NAD O2D	C7–NAD C4N
WT, 4DWV[b]	pentafluoro	NAD^+	1.97 ± 0.01	2.50 ± 0.02	2.65 ± 0.03	3.36 ± 0.04
H51Q, 8G4V	pentafluoro	NAD^+	1.92	2.60	2.68	3.32
WT, 7K35[c]	4-methyl	NADH	2.18 ± 0.03	2.69 ± 0.05	2.74 ± 0.05	3.97 ± 0.09
WT, 7JQA[c]	4-bromo	NADH	2.24 ± 0.04	2.58 ± 0.11	2.68 ± 0.05	3.98 ± 0.16
H51Q/K228R, 1QV6[d]	2,4-difluoro	NADH	2.23	2.68	2.88	4.00
H51Q/K228R, 1QV7[d]	2,3-difluoro	NADH	2.20	2.79	2.84	3.85

[a] "O1" is the oxygen of the alcohol. "C7" refers to the methylene carbon that could donate or receive hydrogen from C4N of the NAD(H). Distances are in Å for the average from two subunits for the enzymes with the H51Q substitutions. The estimated errors (reported in the original publications for the wild-type enzymes) suggest that the Zn–O1 and C7–NAD C4N distances are significantly different in the enzymes with NAD^+ or NADH. [b] For two subunits (one ADH dimer) with NAD^+ and 2,3,4,5,6-pentafluorobenzyl alcohol determined at 1.1 Å after refinement with SHELXL.[5] See also data for complexes of four substituted enzymes (S48T, F57F, F93W, and V203A) complexed with NAD^+ and pentafluorobenzyl alcohol determined at 1.14 Å.[31] [c] For eight subunits of the two structures with NADH and 4-methylbenzyl alcohol determined at 1.2 Å or with 4-bromobenzyl alcohol determined at 1.53 Å.[7] [d] For two subunits after further refinement of structures at 1.8 Å.[1]

Ionization of the Zinc Ligands Are Most Relevant for pH Dependencies of H51Q ADH. The H51Q substitution appears to affect the ionization of the zinc–water and substrates or inhibitors that bind to the catalytic zinc. The only other potential ionizable group with a pK in the range from 6.0–10.5 in the active site of H51Q ADH is Lys-228, which interacts with Asp-223 and the 3'-hydroxyl group and phosphate of the AMP moiety of NAD.[5, 32] Deprotonation of some group with a pK value of 10.4 (a typical pK for lysine residues) in the apoenzyme decreases the affinity for coenzymes and might be attributed to Lys-228[33], but its pK could be increased in the enzyme–NAD^+ complex by electrostatic interactions. Ionization of a group with a pK of 11.2 decreases the affinity for NADH and is assigned to the zinc-water.[33] The H51Q and doubly-substituted H51Q/K228R ADHs have similar pH dependences for the formation of the ternary complexes with NAD^+ and pyrazole (pK values 8.0 and 9.0).[30] It appears that only the water and other ligands bound to the catalytic zinc are involved in the observed pH dependencies for H51Q ADH, but Lys-228 might ionize at pH >10 in some enzyme forms, and involvement of distal amino acids cannot be excluded.

Table S11. pH Dependences of Kinetic Constants for Forward and Reverse Reactions of Horse Wild-type and H51Q ADHs[a]

	Wild-type ADH1E[b]		H51Q ADH[c]	
Kinetic constant	pK values	Limiting values	pK values	Limiting values
NAD^+ and ethanol				
V_1/E_t (s^{-1})	6.5 ± 0.3	k_b 4.1 ± 0.3	7.6 ± 0.3[d]	k_a 0.25 ± 0.05
			9.3 ± 0.4[d]	k_b 3 ± 1
				k_c 10 ± 2
V_1/E_tK_b ($mM^{-1}s^{-1}$)	6.7 ± 0.1	k_m 22 ± 1[e]	8.4 ± 0.2	k_b 11 ± 2
	9.0 ± 0.1			
$k_{H\ ethanol\ burst}$ (s^{-1})	6.4	k_b 155	7.8 ± 0.1[d]	k_a 20 ± 2
			9.6 ± 0.1[d]	k_b 93 ± 6
$K_{i\ CF3CH2OH}$ (μM)	7.6 ± 0.1	K_{db} 4.0 ±0.1	8.5 ± 0.1	K_{db} 4.1 ± 0.2
$K_{i\ pyrazole}$ (μM)	7.24 ± 0.01	K_{da} 270 ± 7	9.0 ± 0.1[e]	K_{da} 150 ± 40
	4.7 ± 0.1	K_{db} 0.7 ± 0.2	7.8 ± 0.2[e]	K_{db} 10 ± 1
K_{ia} (μM)	7.5	K_{da} 220	pH independent	270
	9.0	K_{db} 7		
$k_{on,NAD+}$ ($\mu M^{-1}s^{-1}$)	6.9 ± 0.2	k_m 2.4 ± 0.4 [f]	ND	1.2 [g]
	9.0 ± 0.1			
NADH and acetaldehyde				
V_2/E_t (s^{-1})	7.8 ± 0.1	k_a 140 ± 10	9.5 ± 0.1	k_a 110 ± 10
V_2/E_tK_p ($mM^{-1}s^{-1}$)	8.6 ± 0.1	k_a 300 ± 20	10.0 ± 0.05	k_a 100 ± 5
$K_{i\ C6H5NHCHO}$ (mM)	10.5 ± 0.1	K_{da} 6.7 ± 0.2	10.7 ± 0.1	K_{da} 0.16 ± 0.01
K_{iq} (μM)	9.2 ± 0.2	K_{da} 0.5	ND	
$k_{on,NADH}$ ($\mu M^{-1}s^{-1}$)	9.2 ± 0.2	k_a 11 ± 1	9.5 ± 0.1	k_a 8 ± 1

[a] The kinetic parameters are defined by the Ordered Bi Bi mechanism: V/E_t, turnover number; V/E_tK_m, catalytic efficiency for substrate; k_H, transient oxidation of ethanol; K_i, inhibition constants; $k_{on,NAD+}$ and $k_{on,NADH}$, rate constants for association for NAD^+ or NADH determined with transient kinetics. The pK values and limiting rate constants were calculated from the fits of the data with the logarithmic forms of the equations derived for a simple pH dependence where two forms of enzyme, E and EH, are related by the pK value, and k_a represents the rate constant for reaction of EH, and k_b represents the kinetic constant for reaction of E. K_{da} or K_{db} represent the dissociation constants at low or high pH. If only one form of enzyme is reacting, the equations are $k_{obs} = k_a/(1 + K/[H^+])$ or $k_{obs} = k_b/(1 + [H^+]/K)$, and if both forms react, the "WAVL" equation applies: $k_{obs} = (k_a + k_bK/[H^+])/(1 + K/[H^+])$. V_1/E_t data were fitted with $k_{obs} = (k_a + k_bK_1/[H^+])/(1 + K_1/H^+] + [H^+]/K_2)$, where k_a and k_b are the activities of monoprotonated and unprotonated forms of enzyme, and pK_1 describes the deprotonation of H_2E, and pK_2 is for deprotonation of HE. A nonlinear least squares program was used for fitting with the logarithmic forms of the equations.[34] [b] Compiled from literature.[1, 15-17, 33, 35-39] ND, not determined. [c] pH dependence graphs and data were reported previously, with the sodium phosphate/pyrophosphate buffer system.[1, 30] [d] These data were fitted with $k_{obs} = (k_aK_1/[H^+] + k_b + k_c[H^+]/K_2)/(1 + K_1/H^+] + [H^+]/K_2)$, where k_a, k_b and k_c are the activities of diprotonated, monoprotonated, and unprotonated forms of enzyme, and pK_1 describes the deprotonation of H_2E, and pK_2 is for deprotonation of HE. The data points do not fit a straight line well. [e] Supporting Information Table S2. [f]The apparent association constant as determined by formation of the enzyme–

NAD–pyrazole complex monitored by absorbance at 293 nm.[37] k_m is the maximum for the bell-shaped dependency: $k_{obs} = k_m/(1 + [H^+]/K_1 + K_2/[H^+])$. [g] In 33 mM Na_2SO_4, with fluorescence signal and trapping with trifluoroethanol at pH 7.4 (Main text Table 3 and Table S5.2a).

Steady-state Kinetics of Horse Liver and Yeast ADHs Also Indicate Isomerization of ADH–NAD^+ Complexes. For the simplest Ordered Bi Bi mechanism, the kinetic constant (V_1/E_tK_a) provides the rate constant for the association of NAD^+ (k_{on}), and V_1K_{ia}/E_tK_a is the rate of dissociation of the enzyme–NAD^+ complex (k_{off}).[40] However careful kinetic studies with wild-type horse ADH found that the calculated rate constant for dissociation is less than the observed turnover number for the reverse reaction with acetaldehyde.[35, 41] This discrepancy can be explained by isomerization of the enzyme–NAD^+ complex, which also explains why the calculated rate constant for association of NAD^+ is less than diffusion controlled. The steady-state kinetics for the H51Q and H51Q/K228R ADHs also show this apparent discrepancy.[30] X-ray crystallography also shows that yeast ADH1 changes conformation when ternary complexes form, and the steady-state kinetics of the yeast H48S, H48Q, H44R/H48Q ADHs are also consistent with an isomerization.[42-47]

The pH dependence of V_1/E_tK_a for wild-type horse ADH is bell-shaped with pK values of 6.4 and 9.6, similar to the values of 6.9 and 9.0 determined by transient kinetics for binding of NAD^+ with trapping by pyrazole.[35, 37] (The pK value of 6.9 might have been missed in other transient kinetic studies because the pH range used was 7 and above.[48, 49]) The H44R yeast ADH1 is homologous to wild-type horse ADH1E (with Arg-47), and NAD^+ binds faster (V_1/E_tK_a) as pH increases and reaches a maximum above a pK of 6.9, whereas affinity for NAD^+ ($1/K_{ia}$) increases above a pK of 9.8.[50] The pH dependence for V_1/E_tK_a for yeast H44R/H48Q ADH (homologous to horse H51Q ADH) shows a pK of 8.0, and K_{ia} is pH independent.[47]

The binding of NAD^+ (V_1/E_tK_a) to horse H51Q/K228R ADH increases with pH with a pK value ~8.5 (Supporting Information Table S12), consistent with most rapid binding of NAD^+ to the apoenzyme with a deprotonated zinc-water. The pH dependence for dissociation of NAD^+ (V_1K_{ia}/E_tK_a) has a similar pH dependence, with a pK of ~8.7, but K_{ia} is essentially pH independent. The common term in the expressions for k_{on} and k_{off} with isomerization of the enzyme–NAD^+ complex (main text Scheme 1) is $k_2/(k_{-1} + k_2)$, which is canceled out in the expression for K_{ia}. The pH dependence can be associated with the isomerization (k_2). The previous simulations of proton release during binding of NAD^+ to wild-type horse ADH suggested that isomerization of the enzyme–NAD^+ was coupled to deprotonation of a group with a pK 7.3, presumed to be the zinc–water and perhaps facilitated by His-51.[15] The ionizations of the zinc-water and His-51 are linked, and a protonated His-51 could stabilize the zinc–hydroxide.

Earlier transient studies with wild-type ADH suggested that ionization of the zinc-water, with a pK 9.2–9.5, decreased the rate of association of NAD^+.[48] The conflicting conclusions might be reconciled by an alternative explanation where the pK of 6.4–6.9 is related to ionization of the zinc-water, and the pK of 9.0–9.6 is for His-51. Ionization of the zinc–water and Lys-228 were also proposed to explain the bell-shaped pH dependence for binding of NAD^+.[37]

Table S12. Steady-State Kinetic Constants for H51Q/K228R ADH at Varied pH[a]

pH	6.17	7.10	8.05	8.71	9.00	9.43	9.89
K_a (μM)	114	75	105	51	77	82	75
K_b (mM)	3.66	2.0	0.95	1.02	1.49	1.36	0.78
K_{ia} (μM)	130	155	315	227	222	219	347
V_1/E_t (s^{-1})	0.095	0.37	1.97	4.65	7.62	9.85	8.18
V_1/E_tK_a ($mM^{-1}s^{-1}$)	0.84	4.9	19	91	99	120	109

[a] Initial velocity studies with varied concentrations of NAD^+ and ethanol were studied in 10 mM $Na_4P_2O_7$, 0.25 mM EDTA, and sufficient H_3PO_4, NaH_2PO_4, and Na_2HPO_4 to produce the desired pH and a final ionic strength of 0.1 at 25 ºC. The data were fitted with the equation for a sequential bi mechanism: $v = V_1AB/(K_{ia}K_b + K_aB + K_bA + AB)$ where A and B are the NAD^+ and ethanol concentrations, K_a and K_b are the corresponding Michaelis constants, K_{ia} is the dissociation constant for NAD^+, and V_1/E_tK_a is an estimate of the rate constant for binding of NAD^+ in the Ordered Bi Bi mechanism.[13, 40] Standard errors ranged from 10–25%. The X-ray structures of this enzyme show that Arg-228 fits well in place of Lys-228, although the affinity for coenzymes is somewhat decreased.[1] These data were collected by Dr. Laurie A. LeBrun.[30] The pK values estimated from the equation $k_{obs} = k_b/(1 + [H^+]/K)$ for kinetic constants (V_1/E_t, V_1/E_tK_b, V_1/E_tK_a , V_1K_{ia}/E_tK_a) are ~ 8.5. When the isomerization of the enzyme–NAD^+ complex is included in the kinetic expressions for binding of NAD^+ (Scheme 1 in the main text), $K_{ia} = k_{-1}k_{-2}/k_1(k_2 + k_{-2})$, V_1/E_tK_a = "k_{on}" $= k_1k_2/(k_{-1} + k_2)$, V_1K_{ia}/E_tK_a = "k_{off}" $= k_{-1}k_2k_{-2}/(k_{-1} + k_2)(k_2 + k_{-2})$.

Review of Kinetic Data in Table S11 on the Effects of the H51Q Substitution on Catalytic Activity. Catalytic efficiency for oxidation of ethanol (V_1/E_tK_b) for wild-type enzyme has a bell-shaped pH dependence with macroscopic pK values of 6.7 and 9.0, whereas H51Q ADH is maximally active above a pK of 8.4, with similar maximum values for both enzymes at optimal pH.[1, 35] For both enzymes, ionization of the zinc-water might facilitate binding of the alcohol, and formation of the alkoxide would facilitate hydride transfer (Main text, Scheme 2). In principle, ionizations of interacting groups are thermodynamically linked, and a protonated imidazole could facilitate ionization of the zinc–water.

The kinetic constant V_1/E_tK_b comprises microscopic rate constants for the binding of alcohol, deprotonation of the enzyme–NAD^+–alcohol complex, hydride transfer and release of acetaldehyde, as well as isomerizations, such as of the enzyme–NAD^+ complex.[47] Deuterium isotope effects for H51Q ADH suggest that hydride transfer is partly rate-limiting at pH 8.[1] The simulations of ethanol oxidation suggest that the microscopic pK of the zinc-bound ethanol is 8.1 (Supporting Information Table 7, "FAX", pK_4). Proton release and hydride transfer have similar macroscopic kinetic constants and could be chemically coupled (Main text, Figure 4 and Supporting Information Table S7, as suggested by simulation of a "concerted" mechanism.). Solvent isotope effects suggest that proton and hydride transfer are coupled or synchronous with a low barrier hydrogen bond between the zinc-bound alcohol and the hydroxyl group of Ser-48 in horse or Thr-45 in yeast ADHs.[29, 46, 51]

The turnover number (V_1/E_t) comprises steps after formation of the ternary enzyme–NAD^+–alcohol complex, the release of NADH, and isomerizations, such as the enzyme–NAD^+ complex (Scheme 1).[1, 47] The almost flat pH dependence for the turnover number of ethanol oxidation by wild-type ADH is due to release of NADH, which is probably controlled by opening of the closed enzyme conformation.[35, 48, 52] In contrast, the pH dependence for H51Q ADH is almost linear (slope of 0.33 for log V_1/E_t versus pH), with (poorly estimated) macroscopic pK values of 7.6 and 9.3, and a maximum value of 10 s^{-1} at high pH. The H/D isotope effects for ethanol oxidation decrease from 2.5 at pH 6.1 to 1.1 at pH 10.6. The isotope effects and rate constants indicate that turnover (2.4 s^{-1} at pH 8) is partly controlled by release of NADH (4.0 s^{-1}) and proton/hydride transfer (transient burst,

14 s^{-1}) in pyrophosphate/phosphate buffers.[1] The complicated pH dependence for V_1/E_t might be explained by partly rate limiting hydrogen transfer at low pH and faster dissociation of NADH at high pH where there is less positive charge on the enzyme due to ionization of Lys-228 or the zinc–water (estimated pK 10.4) to bind the negatively-charged NADH. Lys-228 might be involved because H51Q/K228R ADH has simple pH dependences with pK values of ~8.5 for V_1/E_t and V_1/E_tK_b (Supporting Information Table S12), and the common step might be controlled by deprotonation of enzyme–NAD^+ complexes. NAD^+ and NADH bind 14-fold less tightly at pH 8 to H51Q/K228R ADH than to wild-type ADH.[30]

The transient oxidation of ethanol (burst reaction) for wild-type horse ADH has a simple pK of 6.4 for the enzyme–NAD^+–alcohol complex, and the H/D isotope effect indicates that hydride transfer is partly rate-limiting.[29, 38, 53, 54] In contrast, the rate constant for the burst reaction for horse H51Q ADH increases with pH, with a “wavy” pH dependence (almost linear) that can be described with pK values of 7.8 and 9.6, increasing to a maximum of 93 s^{-1}, as compared to a maximum of 155 s^{-1} at pH 8 for wild-type ADH.[38] An alternative explanation for the pH dependence is that base catalysis by hydroxide ion contributes at higher pH (see the main text).

For the reverse reaction, the rate constant for association of NADH to wild-type horse ADH is fastest at low pH, with a pK value of ~9.2, assigned to the zinc-water in the free enzyme.[35, 48, 49] The pH dependences with the H51Q and H51Q/K228R ADHs have pK values of 9.5 and 9.9, consistent with the involvement of the zinc–water.[30] Catalytic efficiency for reduction of acetaldehyde (V_2/E_tK_p) is fastest at low pH, below a pK of 8.6 for wild-type ADH and 10.0 for H51Q ADH, consistent with pK values for zinc–water in the enzyme–NADH complex and some effect on catalytic steps.

Pyrazole binds most rapidly to the wild-type enzyme–NAD^+ complex at low pH and slower above an apparent pK of 7.6, which is attributed to ionization of the enzyme–NAD^+–water.[17] The rate constant for dissociation of pyrazole increases in direct proportion to hydrogen ion concentration over the range pH 4 to 8 with a pK <4. Those data suggest that pyrazole binds most rapidly when the zinc–water is protonated, and the ternary complex is most stable at high pH where pyrazole forms the partial covalent bond with the nicotinamide ring (N2–C4N, 1.6 Å). However, the alternative pathways for binding of a ligand to the protonated enzyme–NAD^+–water to form the enzyme–NAD^+–deprotonated ligand are thermodynamically the same. Transient proton release studies at pH 8 are consistent with 12-fold faster binding of pyrazole to the unprotonated wild-type enzyme–NAD^+ complex and a pK value of 4.7 for the ternary enzyme–NAD^+–pyrazole complex.[15] The steady-state kinetics of inhibition of ethanol oxidation by pyrazole (Supporting Information Table S3) provides an estimated pK of ~9.0 for the H51Q ADH–NAD^+ complex (Main text Table 7). Simulations of the binding of pyrazole and proton release suggest that the pK of the H51Q ADH–NAD^+–pyrazole complex is ~5.7 (Main text Table 6).

Competitive inhibition by trifluoroethanol provides a pK for the wild-type enzyme–NAD^+–water complex of 7.6, and a pK of 4.3 is estimated for the enzyme–NAD^+–trifluoroethanol complex because the complex dissociates most rapidly at low pH.[39, 55, 56] Transient kinetics studies of proton release and quenching of protein fluorescence give similar pK values of 7.3 and 4.6.[15] Note that the pK of 2,2,2-trifluoroethanol of 12.4 in water is decreased to 4.3 in the ternary complex.[54] A pK of 5.8 is estimated for the ternary complex from the simulations (Supporting Information Table S5.3b).

The pH dependence for the binding of *N*-cyclohexylformamide, a competitive inhibitor against acetaldehyde, shows tightest binding at low pH, with pK values of ~10.6 for both enzymes, consistent with decreased affinity for the enzyme–NADH complex with the zinc-hydroxide. *N*-cyclohexylformamide binds 24-fold tighter to wild-type enzyme, indicating different energetics in the hydrogen bond system, although the substrate binding sites are similar – see 5VL0.pdb or 1LDY.pdb for wild-type and 8G39.pdb for H51Q ADH. The turnover numbers (V_2/E_t) are fastest at low pH, with

a pK value of 7.8 for wild-type ADH and 9.5 for H51Q ADH, which can be controlled by the isomerization of the enzyme-NAD^+ complex and release of NAD^+ (Supporting Information Table S5.2a), which is also the case with wild-type enzyme.[35, 41, 52]

General Importance of the Proton Relay System. Although His-51 is a common feature of alcohol dehydrogenases, several ADHs have threonine, serine or tyrosine at that position.[57, 58] The T51S or T51H (actually Thr-52 in the structure, 3COS.pdb) substitutions in human ADH2 cause small changes in enzymatic activity.[59] Perhaps His-48, which binds the NAD O2A can swing around to participate in catalysis.[45] The N51H substitution in mouse ADH2 also has small effects on activity.[60] Mouse ADH2 has Pro-47 (corresponding to Arg-47 in horse ADH), and substitutions P47A, P47Q and P47H increase activity more than 10-fold on benzyl alcohol, but the pH dependencies and isotope effects are similar for all three enzymes, suggesting that hydride transfer is rate-limiting.[61] His-51 is not an absolute requirement for horse liver ADH activity, but it does contribute to proton transfers.

Ser-48 and the homologous threonine in the proton relay system are highly conserved and are very important for catalysis by alcohol dehydrogenases.[57] The S48T substitution in horse liver ADH1E has modest effects on activity, but the S48A substitution decreases the turnover number for oxidation of ethanol by 200-fold and catalytic efficiency by 7 x 10^5-fold, suggesting that proton transfer is blocked and binding of substrate is affected.[62] The homologous T48A substitution in human liver ADH1B inactivates the enzyme.[63] The T45A or T45G substitutions of the homologous Thr-45 in yeast ADH1 decrease turnover with ethanol from 400 s^{-1} to 0.016 s^{-1} or 0.18 s^{-1} respectively.[64] The pH dependence for oxidation of ethanol by T45G ADH (V_1/E_tK_b) shifts from 7.7 to 6.3, and the binding of NAD^+ ($1/K_{ia}$) becomes pH independent.[64] Yeast ADH changes conformation when it binds NADH or forms a ternary complex, but the kinetics of the isomerization and the contribution of His-48 are not known.[42-44]

Future Investigations. The kinetics and structural studies on the wild-type and substituted horse liver and yeast ADHs offer a good basis for modern computational studies that might explain several aspects of the enzyme mechanism. The energetics of the conformational change of horse ADH are associated with domain motions, and effects of substitutions of His-51, Lys-228, Val-292, and Gly293/Pro-295 in complexes with coenzymes and ligands might be quantifiable.[30, 51, 65-67] Two different mechanisms for the exchange of ligands on the catalytic zinc have been proposed.[43, 44, 68] The enzyme–coenzyme complex with the zinc–water (or hydroxide) could form an adjacent, penta-coordinated zinc intermediate with the ligand and then water dissociates. Alternatively, a double displacement mechanism where the zinc–water (or hydroxide) is displaced from the zinc by the nearby Glu-68 to form an intermediate enzyme species with an inverted coordination of the catalytic zinc, followed by displacement of Glu-68 by the ligand.[68] Several alcohol dehydrogenases -- human ADH3, *E coli* ADH, and yeast ADH1 -- have the alternative coordination in apoenzyme or enzyme–coenzyme complexes and also the “classical” coordination in ternary complexes where the ligand has displaced the glutamate residue.[42-44, 69-71] The mechanism for ligand exchange might involve transient protonation of the hydroxide and dissociation of water. The mechanism for binding and deprotonation of pyrazole and formation of the partial covalent bond with NAD^+ is not known. The ionization of the zinc-water and His-51 in the proton relay system are linked, and microscopic pK values for various enzyme complexes could be assigned with calculations using the AMOEBA polarizable force field.[72-75] The pK values and contributions to structure and catalysis of buried ionizable groups, such as Asp-49, Glu-68, and Glu-267, are not esrtablished.[5, 76] Alcohol dehydrogenases have been extensively studied, but many questions remain.

References

(1) LeBrun, L. A., Park, D. H., Ramaswamy, S., and Plapp, B. V. (2004) Participation of histidine-51 in catalysis by horse liver alcohol dehydrogenase. *Biochemistry 43*, 3014-3026.

(2) Pflugrath, J. W. (1999) The finer things in X-ray diffraction data collection. *Acta Crystallogr. D55*, 1718-1725.

(3) Rubach, J. K., and Plapp, B. V. (2003) Amino acid residues in the nicotinamide binding site contribute to catalysis by horse liver alcohol dehydrogenase. *Biochemistry 42*, 2907-2915.

(4) Winn, M. D., Ballard, C. C., Cowtan, K. D., Dodson, E. J., Emsley, P., Evans, P. R., Keegan, R. M., Krissinel, E. B., Leslie, A. G., McCoy, A., McNicholas, S. J., Murshudov, G. N., Pannu, N. S., Potterton, E. A., Powell, H. R., Read, R. J., Vagin, A., and Wilson, K. S. (2011) Overview of the CCP4 suite and current developments. *Acta Crystallogr. D67*, 235-242.

(5) Plapp, B. V., and Ramaswamy, S. (2012) Atomic-resolution structures of horse liver alcohol dehydrogenase with NAD^+ and fluoroalcohols define strained Michaelis complexes. *Biochemistry 51*, 4035-4048.

(6) Plapp, B. V., Savarimuthu, B. R., Ferraro, D. J., Rubach, J. K., Brown, E. N., and Ramaswamy, S. (2017)

(7) Plapp, B. V., and Subramanian, R. (2021) Alternative binding modes in abortive NADH-alcohol complexes of horse liver alcohol dehydrogenase. *Arch. Biochem. Biophys. 701*, 108825.

(8) Jones, T. A., Zou, J. Y., Cowan, S. W., and Kjeldgaard, M. (1991) Improved methods for building protein models in electron density maps and the location of errors in these models. *Acta Crystallogr. A47*, 110-119.

(9) Williams, C. J., Headd, J. J., Moriarty, N. W., Prisant, M. G., Videau, L. L., Deis, L. N., Verma, V., Keedy, D. A., Hintze, B. J., Chen, V. B., Jain, S., Lewis, S. M., Arendall, W. B., 3rd, Snoeyink, J., Adams, P. D., Lovell, S. C., Richardson, J. S., and Richardson, D. C. (2018) MolProbity: More and better reference data for improved all-atom structure validation. *Protein Sci. 27*, 293-315.

(10) Merritt, E. A. (1999) Comparing anisotropic displacement parameters in protein structures. *Acta Crystallogr. D55*, 1997-2004.

(11) Ramaswamy, S., Scholze, M., and Plapp, B. V. (1997) Binding of formamides to liver alcohol dehydrogenase. *Biochemistry 36*, 3522-3527.

(12) Eklund, H., Samama, J. P., and Wallen, L. (1982) Pyrazole binding in crystalline binary and ternary complexes with liver alcohol dehydrogenase. *Biochemistry 21*, 4858-4866.

(13) Cleland, W. W. (1979) Statistical analysis of enzyme kinetic data. *Methods Enzymol. 63*, 103-138.

(14) Theorell, H., Yonetani, T., and Sjöberg, B. (1969) On the effects of some heterocyclic compounds on the enzymic activity of liver alcohol dehydrogenase. *Acta Chem. Scand. 23*, 255-260.

(15) Kovaleva, E. G., and Plapp, B. V. (2005) Deprotonation of the horse liver alcohol dehydrogenase-NAD^+ complex controls formation of the ternary complexes. *Biochemistry 44*, 12797-12808.

(16) Eftink, M. R., and Bystrom, K. (1986) Studies of the pH dependence of the formation of binary and ternary complexes with liver alcohol dehydrogenase. *Biochemistry 25*, 6624-6630.

(17) Andersson, P., Kvassman, J., Lindström, A., Oldén, B., and Pettersson, G. (1981) Effect of pH on pyrazole binding to liver alcohol dehydrogenase. *Eur. J. Biochem. 114*, 549-554.

(18) Theorell, H., and Tatemoto, K. (1971) Excitation transfer in complexes of horse liver alcohol dehydrogenase. *Arch. Biochem. Biophys. 142*, 69-82.

(19) Shore, J. D., Gutfreund, H., and Yates, D. (1975) Quenching of protein fluorescence by transient intermediates in the liver alcohol dehydrogenase reaction. *J. Biol. Chem. 250*, 5276-5277.

(20) Coates, J. H., Hardman, M. J., Shore, J. D., and Gutfreund, H. (1977) Pressure relaxation studies of isomerisations of horse liver alcohol dehydrogenase linked to NAD+ binding. *FEBS Lett. 84*, 25-28.

(21) Wolfe, J. K., Weidig, C. F., Halvorson, H. R., and Shore, J. D. (1977) pH-dependent conformational states of horse liver alcohol dehydrogenase. *J. Biol. Chem. 252*, 433-436.

(22) Abdallah, M. A., Biellmann, J. F., Wiget, P., Joppich-Kuhn, R., and Luisi, P. L. (1978) Fluorescence quenching and energy transfer in complexes between horse-liver alcohol dehydrogenase and coenzymes. *Eur. J. Biochem. 89*, 397-405.

(23) Parker, D. M., Hardman, M. J., Plapp, B. V., Holbrook, J. J., and Shore, J. D. (1978) pH-Dependent changes of intrinsic fluorescence of chemically modified liver alcohol dehydrogenases. *Biochem. J. 173*, 269-275.

(24) Ross, J. B., Schmidt, C. J., and Brand, L. (1981) Time-resolved fluorescence of the two tryptophans in horse liver alcohol dehydrogenase. *Biochemistry 20*, 4369-4377.
(25) Eftink, M. R., and Hagaman, K. A. (1986) Fluorescence lifetime and anisotropy studies with liver alcohol dehydrogenase and its complexes. *Biochemistry 25*, 6631-6637.
(26) Strasser, F., Dey, J., Eftink, M. R., and Plapp, B. V. (1998) Activation of horse liver alcohol dehydrogenase upon substitution of tryptophan 314 at the dimer interface. *Arch. Biochem. Biophys. 358*, 369-376.
(27) Eftink, M. R. (1986) Quenching of the intrinsic fluorescence of liver alcohol dehydrogenase by the alkaline transition and by coenzyme binding. *Biochemistry 25*, 6620-6624.
(28) Laws, W. R., and Shore, J. D. (1978) The mechanism of quenching of liver alcohol dehydrogenase fluorescence due to ternary complex formation. *J. Biol. Chem. 253*, 8593-8597.
(29) Sekhar, V. C., and Plapp, B. V. (1990) Rate constants for a mechanism including intermediates in the interconversion of ternary complexes by horse liver alcohol dehydrogenase. *Biochemistry 29*, 4289-4295.
(30) LeBrun, L. A., and Plapp, B. V. (1999) Control of coenzyme binding to horse liver alcohol dehydrogenase. *Biochemistry 38*, 12387-12393.
(31) Kim, K., and Plapp, B. V. (2020) Substitutions of amino acid residues in the substrate binding site of horse liver alcohol dehydrogenase have small effects on the structures but significantly affect catalysis of hydrogen transfer. *Biochemistry 59*, 862-879.
(32) Ramaswamy, S., Eklund, H., and Plapp, B. V. (1994) Structures of horse liver alcohol dehydrogenase complexed with NAD+ and substituted benzyl alcohols. *Biochemistry 33*, 5230-5237.
(33) Andersson, P., Kvassman, J., Lindström, A., Oldén, B., and Pettersson, G. (1981) Effect of NADH on the pKa of zinc-bound water in liver alcohol dehydrogenase. *Eur. J. Biochem. 113*, 425-433.
(34) Johnson, M. L., and Frasier, S. G. (1985) Nonlinear least-squares analysis. *Methods Enzymol. 117*, 301-342.
(35) Dalziel, K. (1963) Kinetic studies of liver alcohol dehydrogenase and pH effects with coenzyme preparations of high purity. *J. Biol. Chem. 238*, 2850-2858.
(36) Cook, P. F., and Cleland, W. W. (1981) pH variation of isotope effects in enzyme-catalyzed reactions. 2. Isotope-dependent step not pH dependent. Kinetic mechanism of alcohol dehydrogenase. *Biochemistry 20,* 1805-1816.
(37) Sekhar, V. C., and Plapp, B. V. (1988) Mechanism of binding of horse liver alcohol dehydrogenase and nicotinamide adenine dinucleotide. *Biochemistry 27*, 5082-5088.
(38) Brooks, R. L., Shore, J. D., and Gutfreund, H. (1972) The effects of pH and temperature on hydrogen transfer in the liver alcohol dehydrogenase mechanism. *J. Biol. Chem. 247*, 2382-2383.
(39) Shore, J. D., Gutfreund, H., Brooks, R. L., Santiago, D., and Santiago, P. (1974) Proton equilibria and kinetics in the liver alcohol dehydrogenase reaction mechanism. *Biochemistry 13*, 4185-4191.
(40) Cook, P. F., and Cleland, W. W. (2007) *Enzyme Kinetics and Mechanism*, Taylor & Francis Group, LLC., New York.
(41) Wratten, C. C., and Cleland, W. W. (1963) Product inhibition studies on yeast and liver alcohol dehydrogenases. *Biochemistry 2*, 935-941.
(42) Savarimuthu, B. R., Ramaswamy, S., and Plapp, B. V. (2014) Yeast alcohol dehydrogenase structure and catalysis. *Biochemistry 53*, 5791-5803.
(43) Plapp, B. V., Charlier, H. A., Jr., and Ramaswamy, S. (2016) Mechanistic implications from structures of yeast alcohol dehydrogenase complexed with coenzyme and an alcohol. *Arch. Biochem. Biophys. 591*, 35-42.
(44) Guntupalli, S. R., Zhuang, L., Chang, L., Plapp, B. V., and Subramanian, R. (2021) Cryo-electron microscopy structures of yeast alcohol dehydogenase. *Biochemistry 60*, 663-677.
(45) Plapp, B. V., Kratzer, D. A., Souhrada, S. K., Warth, E., and Jacobi, T. (2023) Specific base catalysis by yeast alcohol dehydrogenase I with substitutions of histidine-48 by glutamate or serine residues in the proton relay system. *Chem.-Biol. Interact. 382*, 110588.
(46) Plapp, B. V. (2024) Solvent isotope and mutageness studies on the proton relay system in yeast alcohol dehydrogenase 1. *Chem.-Biol. Interact. 388*, 110853.

(47) Jacobi, T., Kratzer, D. A., and Plapp, B. V. (2024) Substitution of both histidines in the active site of yeast alcohol dehydrogenase 1 exposes underlying pH dependencies. *Chem.-Biol. Interact. 394*, 110992.
(48) Kvassman, J., and Pettersson, G. (1979) Effect of pH on coenzyme binding to liver alcohol dehydrogenase. *Eur. J. Biochem. 100*, 115-123.
(49) DeTraglia, M. C., Schmidt, J., Dunn, M. F., and McFarland, J. T. (1977) Liver alcohol dehydrogenase-coenzyme reaction rates. *J. Biol. Chem. 252*, 3493-3500.
(50) Gould, R. M., and Plapp, B. V. (1990) Substitution of arginine for histidine-47 in the coenzyme binding site of yeast alcohol dehydrogenase I. *Biochemistry 29*, 5463-5468.
(51) Ramaswamy, S., Park, D. H., and Plapp, B. V. (1999) Substitutions in a flexible loop of horse liver alcohol dehydrogenase hinder the conformational change and unmask hydrogen transfer. *Biochemistry 38*, 13951-13959.
(52) Theorell, H., and Chance, B. (1951) Studies on liver alcohol dehydrogenase: II.The kinetics of the compound of horse liver alcohol dehydrogenase and reduced diphosphopyridine nucleotide. *Acta Chem. Scand. 5*, 1127-1144.
(53) Brooks, R. L., and Shore, J. D. (1971) Effect of substrate structure on the rate of the catalytic step in the liver alcohol dehydrogenase mechanism. *Biochemistry 10*, 3855-3858.
(54) Kvassman, J., Larsson, A., and Pettersson, G. (1981) Substituent effects on the ionization step regulating desorption and catalytic oxidation of alcohols bound to liver alcohol dehydrogenase. *Eur. J. Biochem. 114*, 555-563.
(55) Andersson, P., Kvassman, J., Oldén, B., and Pettersson, G. (1984) Synergism between coenzyme and alcohol binding to liver alcohol dehydrogenase. *Eur. J. Biochem. 144*, 317-324.
(56) Kvassman, J., and Pettersson, G. (1980) Effect of pH on the binding of decanoate and trifluoroethanol to liver alcohol dehydrogenase. *Eur. J .Biochem. 103*, 557-564.
(57) Sun, H. W., and Plapp, B. V. (1992) Progressive sequence alignment and molecular evolution of the Zn-containing alcohol-dehydrogenase family. *J. Mol. Evol. 34*, 522-535.
(58) Ramaswamy, S., El-Ahmad, M., Danielsson, O., Jörnvall, H., and Eklund, H. (1996) Crystal structure of cod liver class I alcohol dehydrogenase: substrate pocket and structurally variable segments. *Protein Sci. 5*, 663-671.
(59) Davis, G. J., Carr, L. G., Hurley, T. D., Li, T. K., and Bosron, W. F. (1994) Comparative roles of histidine 51 in human beta 1 beta 1 and threonine 51 in pi pi alcohol dehydrogenases. *Arch. Biochem. Biophys. 311*, 307-312.
(60) Svensson, S., Strömberg, P., and Höög, J.-O. (1999) A novel subtype of class II alcohol dehydrogenase in rodents. Unique Pro-47 and Ser-182 modulates hydride transfer in the mouse enzyme. *J. Biol. Chem. 274*, 29712-29719.
(61) Strömberg, P., Svensson, S., Berst, K. B., Plapp, B. V., and Höög, J.-O. (2004) Enzymatic mechanism of low-activity mouse alcohol dehydrogenase 2. *Biochemistry 43*, 1323-1328.
(62) Kim, K., and Plapp, B. V. (2016) Inversion of substrate stereoselectivity of horse liver alcohol dehydrogenase by substitutions of Ser-48 and Phe-93. *Chem.-Biol. Interact. 276*, 77-87.
(63) Höög, J.-O., Eklund, H., and Jörnvall, H. (1992) A single-residue exchange gives human recombinant ββ alcohol dehydrogenase γγ isozyme properties. *Eur. J. Biochem. 205*, 519-526.
(64) Pal, S., and Plapp, B. V. (2021) The Thr45Gly substitution in yeast alcohol dehydrogenase substantially decreases catalysis, alters pH dependencies, and disrupts the proton relay system. *Chem.-Biol. Interact. 349*, 109650.
(65) Colonna-Cesari, F., Perahia, D., Karplus, M., Eklund, H., Brändén, C.-I., and Tapia, O. (1986) Interdomain motion in liver alcohol dehydrogenase. Structural and energetic analysis of the hinge bending mode. *J. Biol. Chem. 261*, 15273-15280.
(66) Rubach, J. K., Ramaswamy, S., and Plapp, B. V. (2001) Contributions of valine-292 in the nicotinamide binding site of liver alcohol dehydrogenase and dynamics to catalysis. *Biochemistry 40*, 12686-12694.
(67) Hayward, S., and Kitao, A. (2006) Molecular Dynamics Simulations of NAD^+-Induced Domain Closure in Horse Liver Alcohol Dehydrogenase. *Biophysical Journal 91*, 1823-1831.
(68) Ryde, U. (1995) On the role of Glu-68 in alcohol dehydrogenase. *Protein Sci. 4*, 1124-1132.

(69) Sanghani, P. C., Bosron, W. F., and Hurley, T. D. (2002) Human glutathione-dependent formaldehyde dehydrogenase. Structural changes associated with ternary complex formation. *Biochemistry 41*, 15189-15194.
(70) Karlsson, A., El-Ahmad, M., Johansson, K., Shafqat, J., Jörnvall, H., Eklund, H., and Ramaswamy, S. (2003) Tetrameric NAD-dependent alcohol dehydrogenase. *Chem.-Biol. Interact. 143-144*, 239-245.
(71) Sanghani, P. C., Davis, W. I., Zhai, L., and Robinson, H. (2006) Structure-function relationships in human glutathione-dependent formaldehyde dehydrogenase. Role of Glu-67 and Arg-368 in the catalytic mechanism. *Biochemistry 45*, 4819-4830.
(72) Luo, J., and Bruice, T. C. (2001) Dynamic structures of horse liver alcohol dehydrogenase (HLADH): Results of molecular dynamics simulations of HLADH-NAD^+-$PhCH_2OH$, HLADH-NAD^+-$PhCH_2O^-$, and HLADH-NADH-PhCHO. *J. Am. Chem. Soc. 123*, 11952-11959.
(73) Agarwal, P. K., Webb, S. P., and Hammes-Schiffer, S. (2000) Computational studies of the mechanism for proton and hydride transfer in liver alcohol dehydrogenase. *J. Am. Chem. Soc. 122*, 4803-4812.
(74) Gogal, R. A., Nessler, A. J., Thiel, A. C., Bernabe, H. V., Corrigan Grove, R. A., Cousineau, L. M., Litman, J. M., Miller, J. M., Qi, G., Speranza, M. J., Tollefson, M. R., Fenn, T. D., Michaelson, J. J., Okada, O., Piquemal, J. P., Ponder, J. W., Shen, J., Smith, R. J. H., Yang, W., Ren, P., and Schnieders, M. J. (2024) Force Field X: A computational microscope to study genetic variation and organic crystals using theory and experiment. *J. Chem. Phys. 161*, 012501.
(75) Thiel, A. C., Speranza, M. J., Jadhav, S., Stevens, L. L., Unruh, D. K., Ren, P., Ponder, J. W., Shen, J., and Schnieders, M. J. (2024) Constant-pH Simulations with the Polarizable Atomic Multipole AMOEBA Force Field. *J. Chem. Theory Comput. 20*, 2921-2933.
(76) Kim, Y. H., Gogerty, D. S., and Plapp, B. V. (2018) Substitutions of a buried glutamate residue hinder the conformational change in horse liver alcohol dehydrogenase and yield a surprising complex with endogenous 3'-dephosphocoenzyme A. *Arch. Biochem. Biophys. 653*, 97-106
